# Supplementary material for: LIFEPLAN: A worldwide biodiversity sampling design
Source: PLoS One. 2024 Dec 31;19(12):e0313353. doi: 10.1371/journal.pone.0313353 (PMC11687782; doi:10.1371/journal.pone.0313353)
Supplement: S1 File — (PDF) [file pone.0313353.s001.pdf]

Dec 05, 2023 Version 3

Lifeplan Camera Trapping Protocol V.3

DOI
dx.doi.org/10.17504/protocols.io.q26g7pxp1gwz/v3

LIFEPLAN
A PLANETARY INVENTORY OF LIFE

Hanna M.K. Rogers[1], Gaia Giedre Banelyte[1], Arielle M Farrell[1], Bess Hardwick[2], Tommi Mononen[2], Deirdre Kerdraon[1]

[1]Department of Ecology, Swedish University of Agricultural Sciences; [2]University of Helsinki

Hanna M.K. Rogers
Department of Ecology, Swedish University of Agricultural Sc...

OPEN ACCESS

DOI: dx.doi.org/10.17504/protocols.io.q26g7pxp1gwz/v3

Protocol Citation: Hanna M.K. Rogers, Gaia Giedre Banelyte, Arielle M Farrell, Bess Hardwick, Tommi Mononen, Deirdre Kerdraon 2023. Lifeplan Camera Trapping Protocol. **protocols.io https://dx.doi.org/10.17504/protocols.io.q26g7pxp1gwz/v3**Version created by **Hanna M.K. Rogers**

License: This is an open access protocol distributed under the terms of the **Creative Commons Attribution License**, which permits unrestricted use, distribution, and reproduction in any medium, provided the original author and source are credited

Protocol status: Working
We use this protocol and it's working

Created: December 04, 2023

Last Modified: December 05, 2023

Protocol Integer ID: 91756

Keywords: Camera trap, Lifeplan, Biodiversity, Camera trapping, Wildlife

Funders Acknowledgement:
European Union's Horizon 2020 research and innovation programme
Grant ID: 856506

Abstract

Lifeplan is a global biodiversity monitoring project with the aim of assessing the current state of biodiversity worldwide, and using this knowledge to generate predictions of how biodiversity might look in the future.

In this protocol we describe the method used within the Lifeplan project for collecting camera trap data on an international scale in a wide variety of environmental conditions and habitats. The aim is to use this data to identify species from images and create species lists for the different locations across the globe. This protocol contains a detailed description from setup of the cameras in the field to weekly data collection, as well as steps to configure cameras and collect necessary information to help in species identification.

We identify the equipment used in Lifeplan, but also give technical specifications of that equipment so that other users of this protocol can find equivalent alternative equipment. We also specify what metadata should be collected with the camera trap data. The technical solution we use to collect metadata in the Lifeplan project is described in detail in the full Lifeplan protocol.

The expected outcome of this method is camera trap images with associated metadata. The species identification process is outside the scope of this protocol and will be described elsewhere.

Image Attribution

Lifeplan

Guidelines

The aim of the method outlined in this protocol is to collect photos of local wildlife across the globe, and collect relevant information that will help in species identification. The ultimate goal is to generate species lists and occurrence data for the local wildlife, and through that get an assessment of the global diversity of terrestrial vertebrates.

To help in species identification it is important to consider the placement of the cameras and to collect information about where and when the photos were taken.

Placement and use of cameras

Environmental conditions and vegetation structures differ wildly across the globe, which makes a standardised placement of the camera traps complicated. There are however several things that you can consider to optimise the detection of animals and to get good quality images for species identification. The first is to carefully examine the ground and structures within the camera's range to see what will affect the image quality and detection rate. The following things should be considered when following the deployment steps in the protocol:

- Some vegetation and/or human structures might seem of low importance in daylight, but can reflect the flash at night and result in images where animals cannot be identified.
- Remember that vegetation height and structure changes over the seasons.
- Vegetation moves in windy conditions, so branches etc. can obscure and/or trigger the camera on windy days.
- A hole or depression in the ground just in front of the camera will cause some animals to pass under the camera lens and not be caught in the images.

Environmental conditions are also important to consider when using the camera traps:

- If the cameras are placed in direct sunlight in places with high temperatures they can become overheated and malfunction. Try to place the cameras in the shade or use a roof to block the sun.
- In cold climates batteries will drain faster and might not last a full week.
- In extreme cold the cameras will not function.

Collecting additional information

The image itself is not always enough to identify a species. Therefore it is important to collect additional information about when and where the image was taken. In Lifeplan we use a custom made app and 6 character QR codes to identify equipment and samples (in this case camera traps and SD cards).

The information collected in the Lifeplan app includes:
- Site ID and who is collecting the data.
- Date and time of sampling event.
- GPS point of where the camera is located.
- QR codes to identify camera trap and SD card.
- Status of equipment and sample.

Aside from the metadata collected through the Lifeplan app the camera traps themselves record the following information:

- Camera ID, to identify which camera has taken the photo.
- Battery status.
- Moon phase.
- Date and time in UTC at which the photo was taken.
- Ambient temperature at the time the photo was taken.

If you are not using the Lifeplan app it is important to **take a separate GPS point** and connect it to the camera ID since the camera traps themselves do not record location.

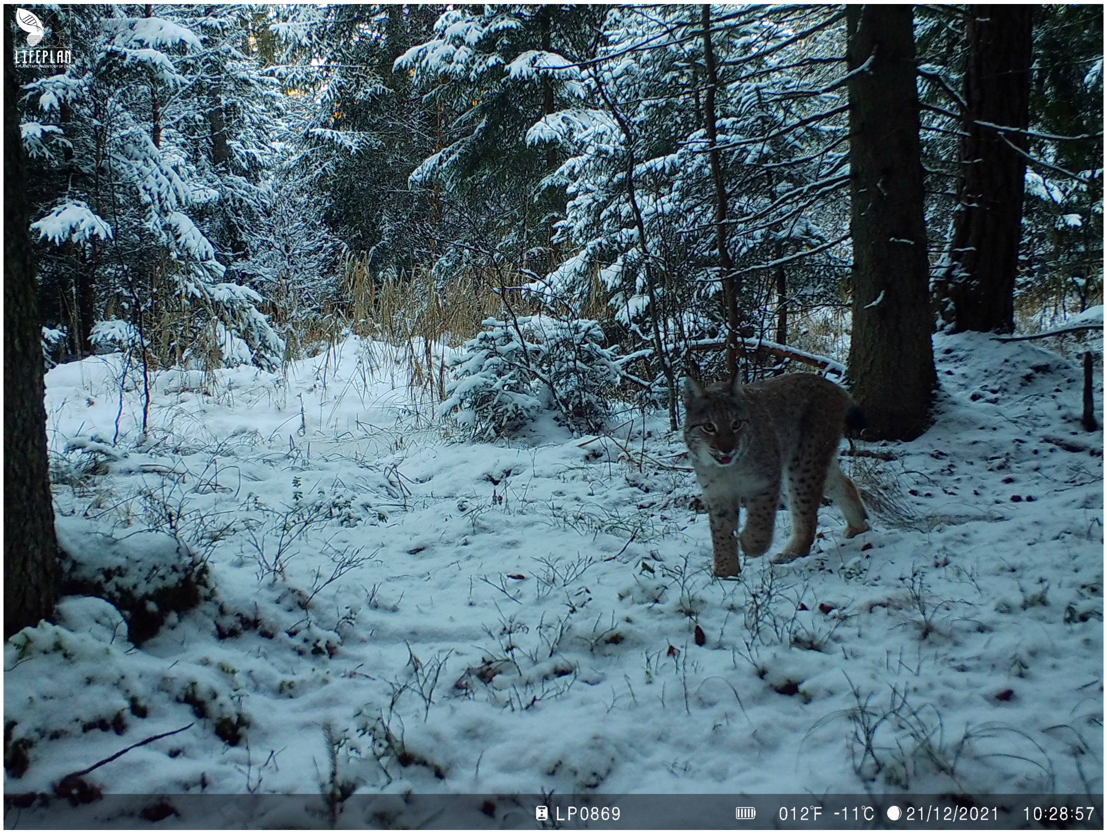

Camera trap photo showing an information bar in the bottom containing important information for species identification: Camera ID, battery status, temperature in Fahrenheit and Celsius, moon phase, and UTC date and time.

Although the cameras use and display time to the second, in practice it may not be correct to the second. This is because setting the date and time manually in the camera introduces some inaccuracy, but also because when taking very large numbers of images the internal time of the cameras may lag slightly over the one-week sampling period. For these reasons, and because we want to be able to sync camera data precisely with our separately collected audio data, we take an image of the precise UTC time at the start and end of the sampling week to calibrate time to the second.

Materials

Required equipment:

- **Camera traps x 3 alt. 5** (Custom Lifeplan model 4.0C from Wildlife Monitoring Solutions)

Equipment	
**Lifeplan Trail Camera**	NAME
Camera Trap	TYPE
Wildlife Monitoring Solutions	BRAND
Lifeplan	SKU
https://www.wildlifemonitoringsolutions.com/	LINK

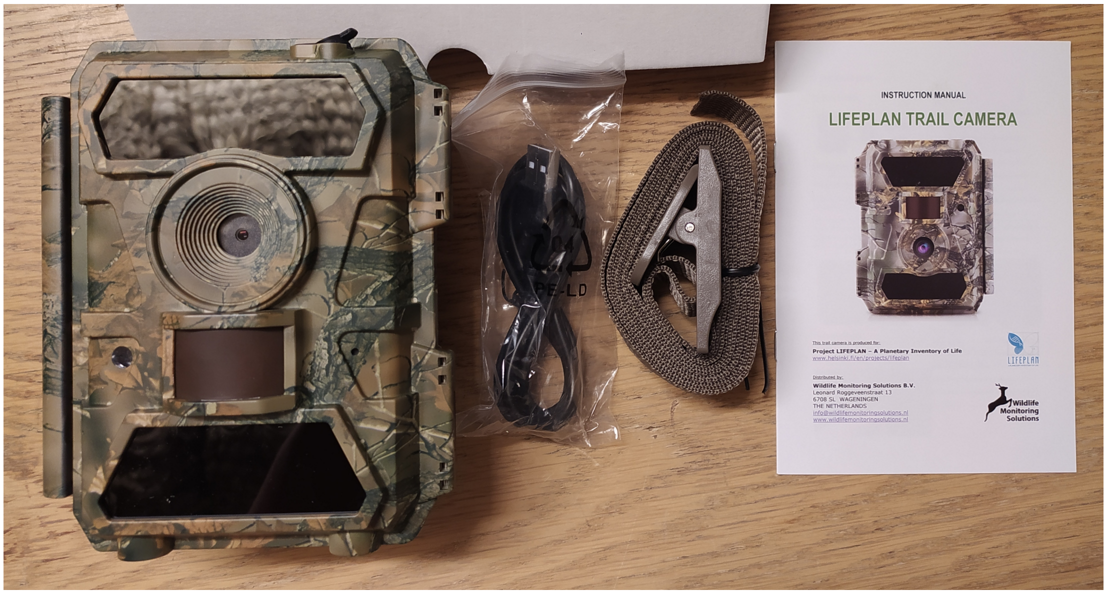

The camera traps are motion triggered using a passive infrared sensor and are set to operate continuously at each sampling plot. When the camera trap is triggered it will take a series of 5 pictures approximately 1s apart. Each image contains an information bar with the camera ID, battery level, temperature in Celsius and Fahrenheit, moon phase, date, and time.
The Lifeplan camera traps are also equipped with a passcode to minimise risk of tampering.

A	B
Image Sensor	5 Mega Pixels Color CMOS
Effective Pixels	2560x1920
Day/Night Mode	Yes
IR range	20m
IR Setting	Top: 27 LED, Bottom: 30 LED
Memory	SD Card (4GB - 32GB)
Lens	F=3.0; FOV=52°; Auto IR-Cut-Remove (at night)
PIR Angle	60°
LCD Screen	2" TFT, RGB, 262k
PIR distance	20m (65 feet)
Picture size	8MP / 12MP / 24MP
Picture Format	JPEG
Video resolution	FHD (1920x1080), HD (1280x720), WVGA (848x480)
Video Format	MOV
Video Length	05-59 sec. programmable
Multi Shot	1-5 pictures per trigger
Trigger Time	0.3s
Trigger Interval	Configurable between 3 seconds and 24 hours
Photo + Video mode	Yes
Time lapse	Yes
SD Card cycle option	Yes
Operation Power	Battery: 9V; DC: 12V
Battery Type	12AA
External DC	12V
Stand-by Current	0.135mA
Stand-by Time	5~8 months (6xAA~12xAA)
Interface	USB/SD Card/DC Port
Mounting	Strap (back); Tripod (bottom and back); Python cable (back)
Operating temperature	-25°C to 60°C
Storage temperature	-30°C to 70°C
Operation humidity	5% - 90%
Waterproof specification	IP66
Dimensions	148*117*78mm
Weight	448 grams
Certifications	CE / FCC / RoHs

Lifeplan camera trap specifications

The general user manual for the Lifeplan camera traps, including default settings:

General_manual_lifeplan_trail_camer… 3.1MB

- **SD-cards** (Sandisk Extreme/Extreme PRO., 32 GB) x 6/10
- **Rechargeable AA batteries** (Eneloop, Ni-MH, 1.2V) x 36/60
- **Silica Gel Desiccant** (RS PRO, 10g packets) x 6/10
- **Battery charger** (Jupio, Master Charger 2) x 1

Optional equipment:

- **Plastic roofs** (handmade from plastic buckets) x 3/5
- **Python cables** or other secure strap x 3/5
- **Solar panels** (WingHome Solar Charger SP100) x 3/5

A	B
Model	Parameters
Built-in Li-ion battery	1500mAH/7.4V
Solar panel max output power	2W
Output Voltage	12V
Max output current	1600mA
Output plug	4.0*1.7*10.0mm(DC2.1)
Power adapter	Input AC110-220, Output:12V 1.0-2.0A
Mounting	Strap/tripod
Water proof	IP54
Operation temperature	-22 - +158F, -30 - +70C
Operation humidity	5% - 95%

Solar panel specifications (Solar Charger SP100)

Before start

Make sure you have obtained the necessary permits for putting up camera traps according to local legislation and land ownership.

Preparing the cameras before deployment

1 **Assigning a unique ID to the camera**

To easily identify which photos originate from which camera, each camera is given a unique ID that is visible on the images and written on the physical camera. Lifeplan cameras have an assigned ID which starts with LP followed by 4 numbers.

Check if the Camera ID registered matches the ID written on the camera:

- Insert 6 charged batteries per camera in the top row or connect the camera to a power source.
- Move the ON/SETUP/OFF switch to SETUP.
- Every time you start the camera you need to enter a passcode. Wait for this screen to pop up.
- Enter the passcode and press OK.
- Press MENU
- Use the arrow keys to select “Cam ID” under the “Cam” tab. Select ON and press OK to see the camera ID.
- Check if it is the same as the ID written on the camera.
- If it is not, change the digital camera ID numbers with the up and down arrows to match the one written on the camera.

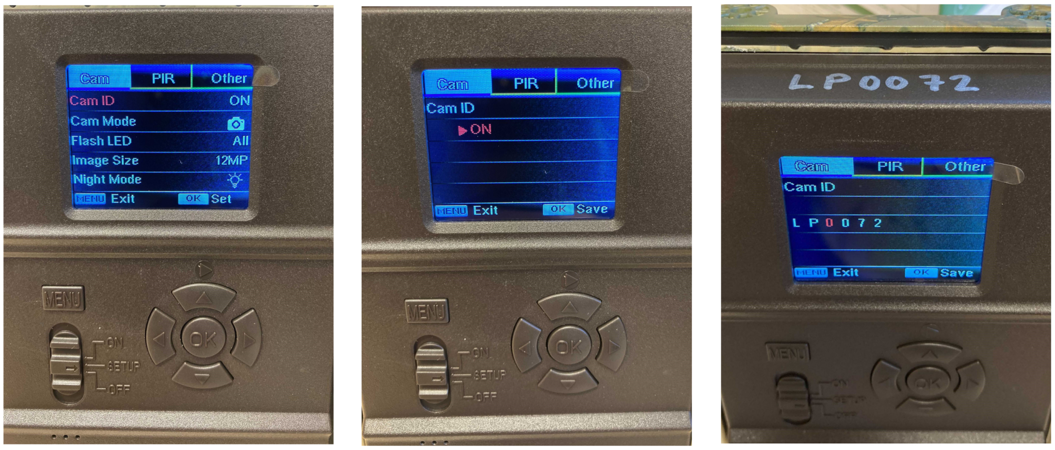

Navigation through the camera menus to access the Cam ID. Last panel shows the Cam ID written on the camera as well as the one entered in the camera's settings.

2 **Change the Night Mode setting:**
The Night Mode setting is changed to reduce blur in the photos, especially at night, and thereby increase the chance of species identification.

- Move the ON/SETUP/OFF switch to SETUP.
- Enter the passcode and press OK.
- Press MENU.
- In the "Cam" tab, use arrow keys to go to "Night Mode". Select "Min.Blur" mode and press OK.
- Set the switch to OFF.

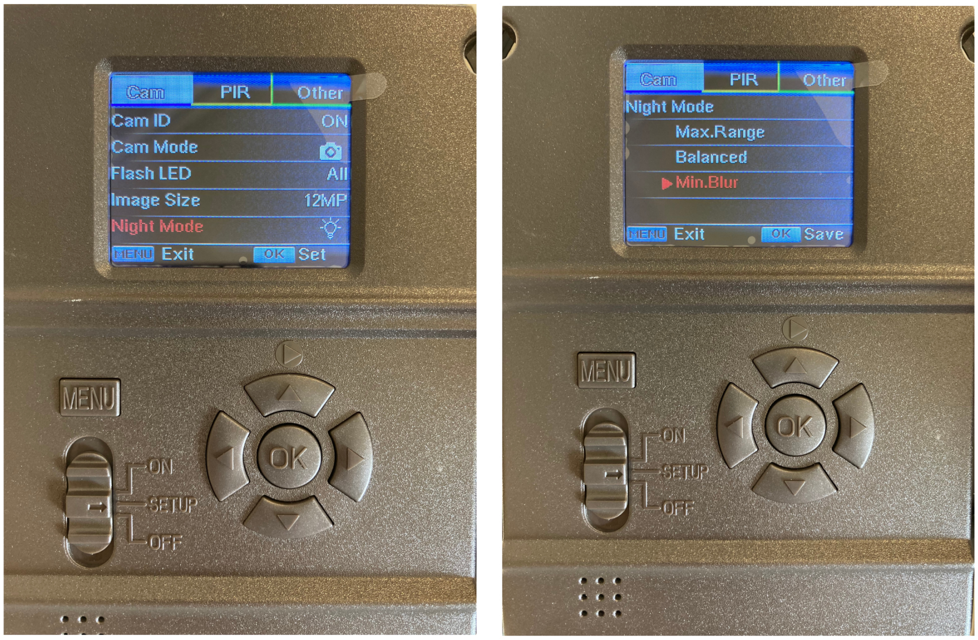

Navigation through the camera menus to access and change the camera's Night mode setting.

3 Set Coordinated Universal Time (UTC)

We set all our cameras to UTC to match the time on the audio recorders (see Lifeplan audio recorder protocol) and so that cameras deployed in different time zones use the same time standard.

NOTE! Without a power source the date and time will reset after a short time. Set the date and time as shortly before deploying the cameras as possible and change the batteries quickly when you perform weekly maintenance.

- Insert 6 fully charged batteries per camera in top row, or connect camera to a power source. The bottom row will be used for a silica gel pouch in wet conditions.
- Move the ON/OFF/SETUP switch to SETUP.
- Enter the passcode and press OK.

- Set time to UTC: Press MENU and navigate to the “Other” tab with arrow keys. Select "Date/Time". Enter UTC date and time in format DD:MM:YYYY, HH:MM:SS. To get UTC time, use the free AudioMoth chime app by Open Acoustic Devices.
- Turn the camera OFF until you place it in the field to save the batteries.

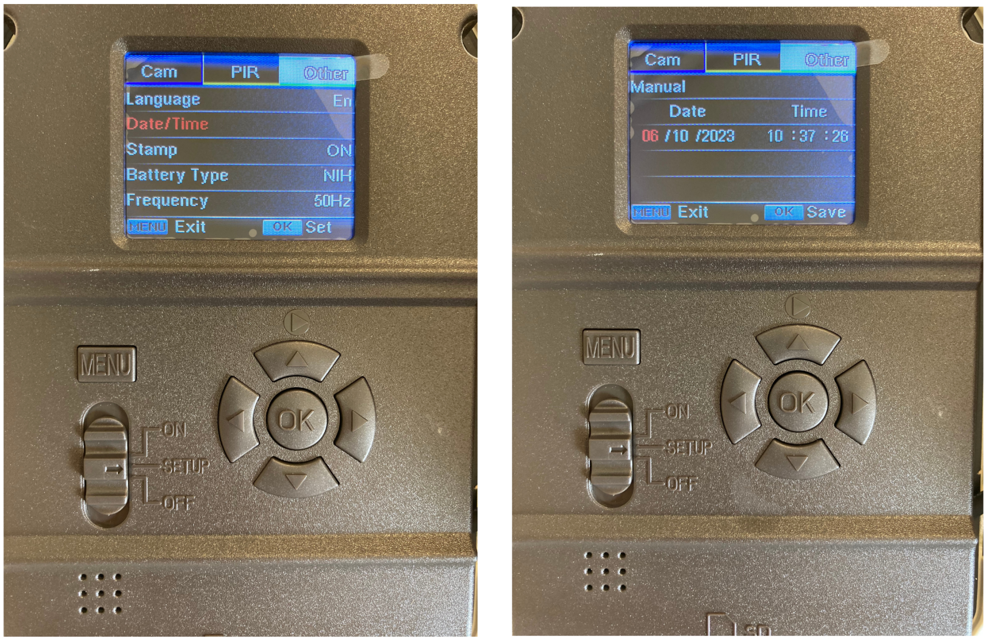

Navigation through the camera menus to access and set the camera's internal date and time.

4 **Organise your SD cards**

For the Lifeplan project, you will have received labels for each SD card with unique 6 letter code starting with the letter "L". This is to facilitate sorting and organising photos that originate from many different cameras.

Attach a label to each SD card. Connect each SD card to a computer and rename them internally with their unique identifier.

Site selection and camera placement

5 **Site design**

The Lifeplan equipment is deployed within a 1 hectare plot. We aim for an accuracy of 10 m. There are two design options to choose from depending on budget and site access. In Lifeplan, we started with Option 1 in 2021, and reduced to Option 2 in 2023 by removing two of the cameras.

Option 1: Four cameras are set at the corners of the 1 hectare square, with a fifth camera in the centre.
Option 2: Three camera traps are set up diagonally within the plot, with about 70 metres between each trap and 140 metres between the farthest traps.

In Lifeplan the corner/outer points are given individual numbers from 1-4, whereas 5 is assigned to the centre.

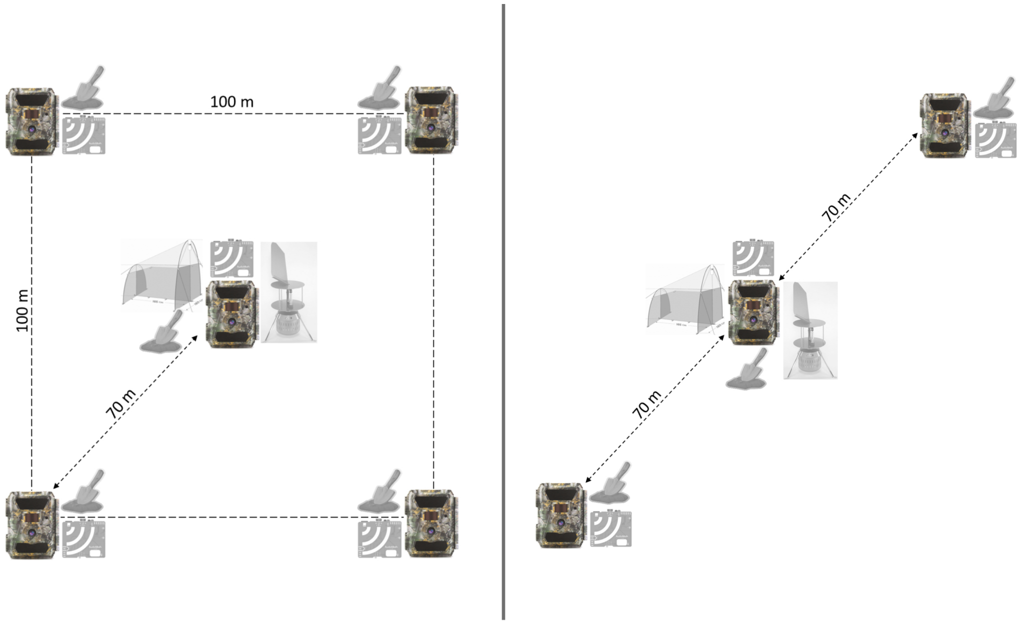

Diagrams of the two different Lifeplan plot designs, with cameras shown in colour and the other Lifeplan equipment shown in grey. Left: Option 1 with five cameras, Right: Option 2 with three cameras.

6 **Tree/post selection**

The camera traps will be mounted on trees or on sturdy posts. The tree/post should be sturdy enough to withstand weather and not sway in windy conditions as this might trigger the camera.

- Select a tree that looks like it will remain standing for the entire study period.
- If you are in an open habitat and put up a post, keep in mind that it might be seen as an excellent scratching post for local wildlife. Make sure the post is sturdy enough and suitable for the environment it is placed in.
- Check the area for local wildlife (such as ant or termite nests) or other environmental factors which could make maintenance difficult.

- For Lifeplan, the audio recorders are mounted on the same trees/posts, so there should be some free space above the cameras.

7 **Deploying cameras:**

Locate a suitable tree/post (see Step 6). Select a place with the least amount of obscuring vegetation in front of the camera and don't point the centre camera at other equipment as this might trigger the camera.

The camera traps should be:

- Mounted at a height of **0.5 metres** from the ground.

- Facing north in the northern hemisphere and south in the southern hemisphere to avoid glare from the sun.

- Perpendicular to the ground in front of the camera. If the tree is located on a slope, the camera will need to be angled either upwards or downwards. If possible, choose to angle the camera downwards to avoid glare from the sun. The angle can be achieved by using sticks or stones found at the site and wedging them between the camera and the tree.

NOTE: If there is snow or growing vegetation which cannot be cut down, move the camera up or down accordingly so it starts each week 0.5 m above the snow or vegetation.

7.1 When you have found suitable locations, fasten the cameras using the straps or python cables.

NOTE: If needed and possible, cut down tall vegetation in front of the camera to increase visibility and to reduce false triggering by vegetation moving in the wind.

It is recommended to put up a roof over the camera to avoid water damage. This can be done with a bucket cut in half.

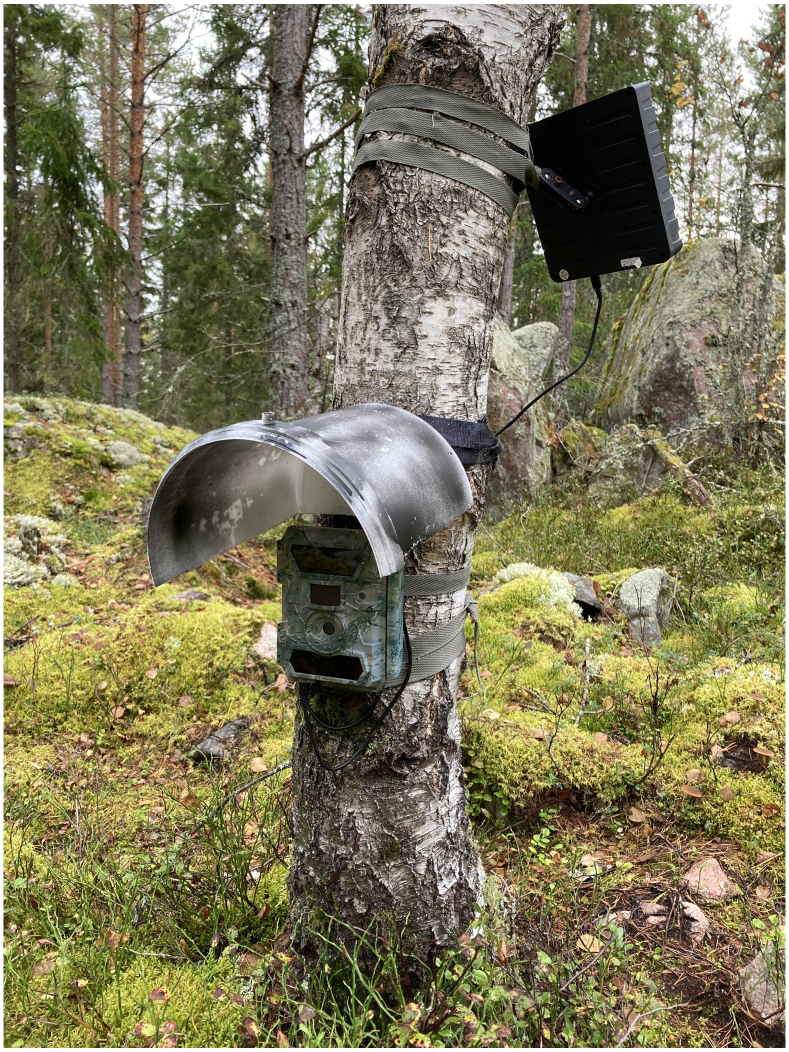

Camera trap with a rain protecting roof. This camera is powered by a solar panel.

7.2 Once you have set up the camera trap, record a GPS location and connect it to the camera ID (you can for example name the GPS point with the camera ID). Write down for yourself where the camera trap is positioned, including in which direction the camera is pointing, so you remember where it is and how it looked in case the camera moves out of position. Also note down if there are any interesting features in the immediate area that might affect detection (for example game trails).

Note! It is important to take a GPS point for each camera and associate the GPS point with the camera ID. This way each photo is linked to a location.

7.3 Put up appropriate signage by the camera traps according to local legislation. Depending on your country and whether the land is private or public, there may be restrictions on placing cameras.

Sampling

8 Sampling is carried out **weekly** to reduce loss of data and to match the sampling schedule of the audio recorders.

Start sampling:

8.1

Open the camera trap.

Check that the camera is OFF.

Insert charged batteries if you have not done it earlier, or connect camera to a power source (for example a solar panel).

8.2 Insert an empty formatted SD card and switch camera to SETUP.
Enter the password and press OK.

8.3 Check that the date and UTC time are correct:

- You should see a live picture on the screen.
- Hold the right arrow button down to see the camera date and time in red text over the screen.
- If incorrect, Press MENU and navigate to “Other” > Date/time to correct it. You can find current UTC time conveniently in the AudioMoth chime app.

8.4 Take a “start picture” to calibrate time:

- Hold your phone screen, with the AudioMoth chime app in Dark Mode, about 20 cm from the lens of the camera. You should be able to see your phone on the camera screen to help position it.
- Press OK to take a picture manually. Try to avoid reflections or glare.
- Quickly check the image by pressing the up arrow. It is okay if the text is blurry: it will be read by a computer.

Note! If you have trouble getting a good image in low light conditions, try lowering the brightness on your phone. The same goes for very bright light conditions, where you can increase the brighness on your phone.

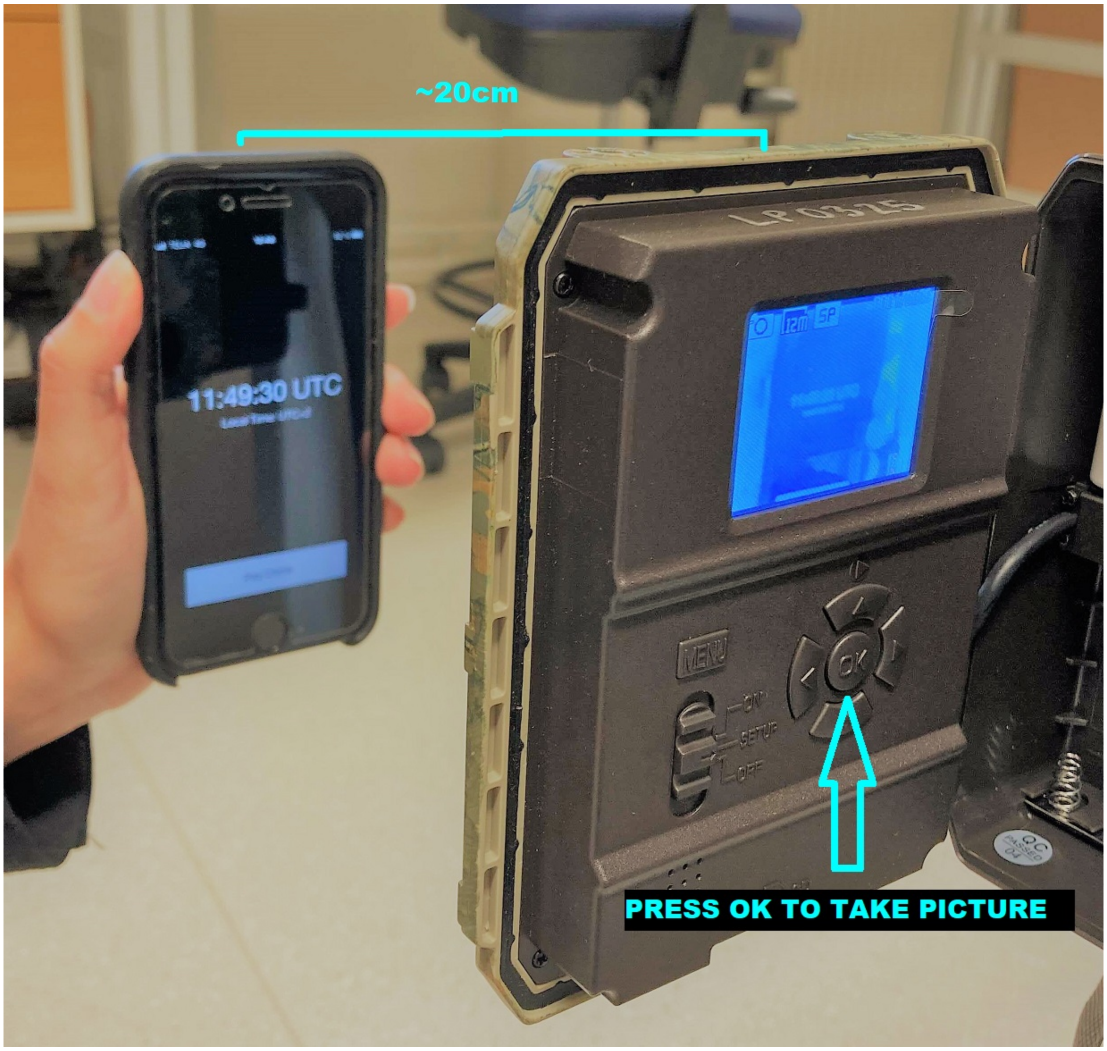

Manually taking a picture of the AudioMoth chime app to establish UTC time to the second.

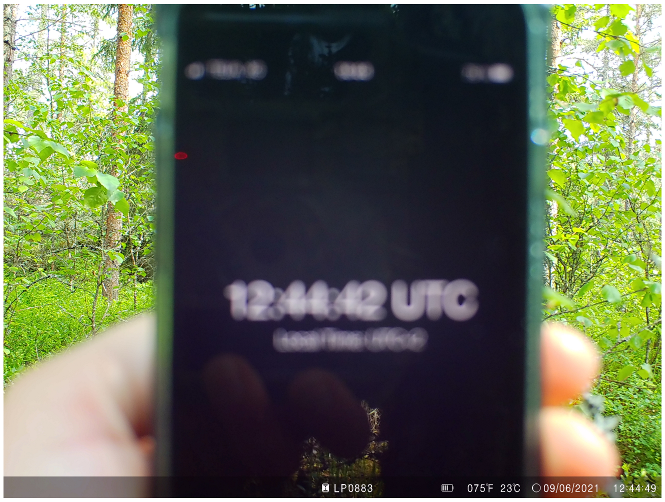

An example of a good image of the AudioMoth chime app.

8.5 Add a dry silica packet in the lower battery row.

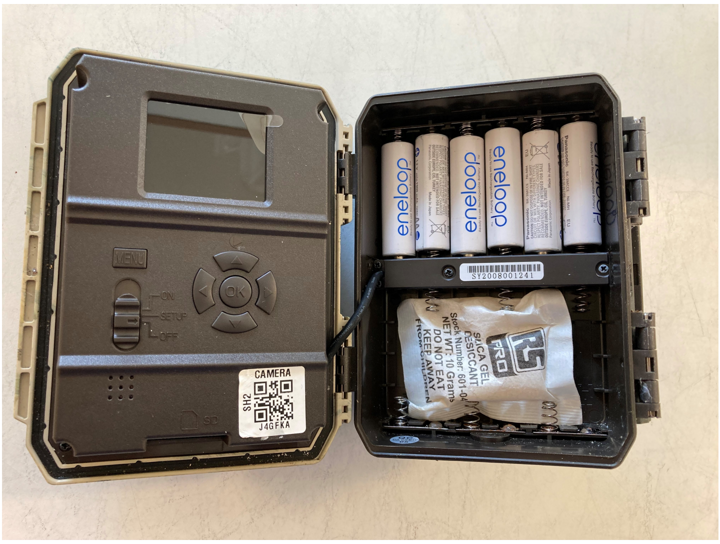

The top row of the battery compartment holds six batteries, and the bottom row is packed with silica gel to remove moisture from inside the camera.

8.6 Switch the camera trap to ON. It is now motion activated and will take 5 picture series of you if you walk in front of it.

Secure the latch.

Note! Be careful to close the camera properly and make sure there is nothing interrupting the seal on the camera, as this can cause moisture to get in and the camera to malfunction.

9 **Collecting the data:**

9.1 Make sure you have enough charged batteries for all the cameras, and that you have a system in place to separate empty batteries from the charged ones.

When arriving at a camera, do a quick scan of the area and the camera trap to check if anything is out of the ordinary. If the camera has moved from its original place (e.g. if it has turned to one side or slipped down), return it to its proper position and make a note of the occurrence.

9.2 Open the camera.

Set the camera to SETUP and enter the password.

9.3 Take an “end picture” to calibrate time (see images in 8.4):

- Hold your phone screen, with the AudioMoth chime app in Dark Mode, about 20 cm from the lens of the camera. You should be able to see your phone on the camera screen to help position it.
- Press OK to take a picture manually. Try to avoid reflection or glare.
- Quickly check the image by pressing the up arrow. It is okay if the text is blurry: it will be read by a computer.

Note! If you have trouble getting a good image in low light conditions, try lowering the brightness on your phone. The same goes for very bright light conditions, where you can increase the brighness on your phone.

9.4 Check if date and UTC time are correct:

- Hold the right arrow button down to see the camera date and time in red text over the screen.
- If incorrect, Press MENU and navigate to “Other” > Date/time to correct it. You can find current UTC time in the AudioMoth chime app.

9.5 Check that the batteries have more than 1 bar of charge. If it is 1 bar or below, turn the camera OFF and change the batteries.

9.6 Switch the camera to OFF**.**

Remove the full SD card and insert an empty formatted card.

9.7 Switch the camera trap to SETUP and enter the password.

Take a “start picture” to calibrate time (see images in 8.4):

- Hold your phone screen, with the AudioMoth chime app in Dark Mode, about 20 cm from the lens of the camera. You should be able to see your phone on the camera screen to help position it.
- Press OK to take a picture manually. Try to avoid reflection or glare.
- Quickly check the image by pressing the up arrow. It is okay if the text is blurry: it will be read by a computer.

Note! If you have trouble getting a good image in low light conditions, try lowering the brightness on your phone. The same goes for very bright light conditions, where you can increase the brighness on your phone.

9.8 Change the silica packet to a dry one as required:

- Every week in wet and humid environments.
- Once a month in dry environments.

9.9 Switch the camera trap to ON and secure the latch.

Note! Be careful to close the camera properly and make sure there is nothing interrupting the seal on the camera, as this can cause moisture to get in and the camera to malfunction.

Preparations for next sampling event

10 Prepare the equipment you need for next week's sampling:

10.1 When you are back from the field transfer the data on the SD cards to your computer and/or external hard drive. Quickly check the images to make sure the SD cards are not malfunctioning.
Format the SD cards before deploying them again.

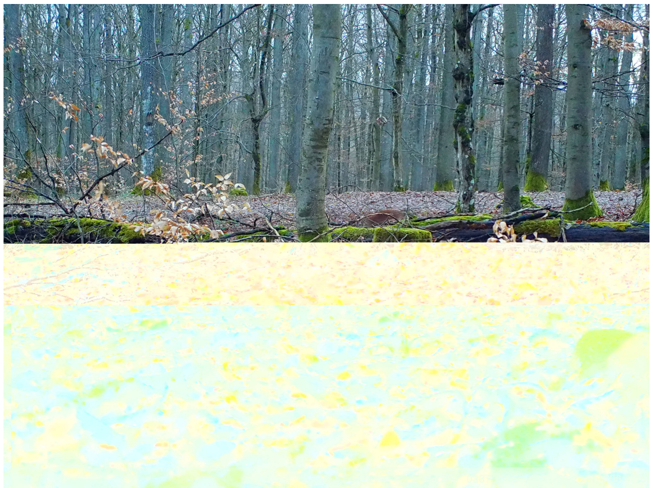

Example of a corrupted image from a malfunctioning SD card.

10.2 Charge batteries for the next week. Charge them as close to changing them in the field as possible to retain maximum amount of charge.

10.3 Dry silica packets
The silica packets can be dried in an oven at maximum 100° C for 40 hours.

Protocol references

Background research & further reading:

Anile, S. & Devillard, S. (2015). Study design and body mass influence RAIs from camera trap studies: evidence from the Felidae. Animal Conservation, 19(1), 35-45. https://doi.org/10.1111/acv.12214

Hofmeester, T.R., Cromsigt, J., Odden, J., Andren, H., Kindberg, J. & Linnell, J.D.C. (2019). Framing pictures: A conceptual framework to identify and correct for biases in detection probability of camera traps enabling multi-species comparison. Ecol Evol, 9(4), 2320-2336. https://doi.org/10.1002/ece3.4878

Jacobs, C.E., Ausband, D.E. & Rowcliffe, M. (2018). An evaluation of camera trap performance – What are we missing and does deployment height matter? Remote Sensing in Ecology and Conservation, 4(4), 352-360. https://doi.org/10.1002/rse2.81

Meek, P.D., Ballard, G., Claridge, A., Kays, R., Moseby, K., O'Brien, T., O'Connell, A., Sanderson, J., Swann, D.E., Tobler, M. & Townsend, S. (2014). Recommended guiding principles for reporting on camera trapping research. Biodiversity and Conservation, 23(9), 2321-2343. https://doi.org/10.1007/s10531-014-0712-8

Meek, P.D., Ballard, G.A., Falzon, G., Rowcliffe, M. & De Angelo, C. (2016). The higher you go the less you will know: placing camera traps high to avoid theft will affect detection. Remote Sensing in Ecology and Conservation, 2(4), 204-211. https://doi.org/10.1002/rse2.28

Sirén, A., Pekins, P., Abdu, P. & Ducey, M. (2016). Identification and Density Estimation of American Martens (Martes americana) Using a Novel Camera-Trap Method. Diversity, 8(4). https://doi.org/10.3390/d8010003

Swann, D.E., Hass, C.C., Dalton, D.C. & Wolf, S.A. (2004). Infrared-triggered cameras for detecting wildlife: an evaluation and review. Wildlife Society bulletin, 32 (2), 357–365. https://doi.org/10.2193/0091-7648(2004)32[357:ICFDWA]2.0.CO;2

Wearn, O.R. & Glover-Kapfer, P. (2017). Camera-trapping for conservation: a guide to best-practices. WWF Conservation Technology Series 1(1). Woking, United Kingdom. https://www.wwf.org.uk/sites/default/files/2019-04/CameraTraps-WWF-guidelines.pdf

Wearn, O.R. & Glover-Kapfer, P. (2019). Snap happy: camera traps are an effective sampling tool when compared with alternative methods. R Soc Open Sci, 6(3), 181748. https://doi.org/10.1098/rsos.181748
