# Supplementary material for: LIFEPLAN: A worldwide biodiversity sampling design
Source: PLoS One. 2024 Dec 31;19(12):e0313353. doi: 10.1371/journal.pone.0313353 (PMC11687782; doi:10.1371/journal.pone.0313353)
Supplement: S2 File — (PDF) [file pone.0313353.s002.pdf]

May 08, 2024 Version 2

Lifeplan Audio Recording Protocol V.2

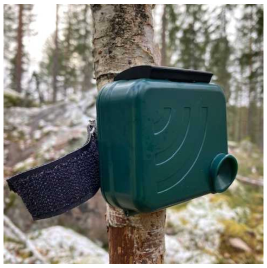

DOI
dx.doi.org/10.17504/protocols.io.kqdg3xbp1g25/v2

Arielle M Farrell[1], Gaia Giedre Banelyte[1], Hanna M.K. Rogers[1], Petteri Lehikoinen[2], Deirdre Kerdraon[1], Bess Hardwick[3]

[1]Department of Ecology, Swedish University of Agricultural Sciences;
[2]The Finnish Museum of Natural History, University of Helsinki; [3]University of Helsinki

Arielle M Farrell
Department of Ecology, Swedish University of Agricultural Sc...

OPEN ACCESS

DOI: dx.doi.org/10.17504/protocols.io.kqdg3xbp1g25/v2

Protocol Citation: Arielle M Farrell, Gaia Giedre Banelyte, Hanna M.K. Rogers, Petteri Lehikoinen, Deirdre Kerdraon, Bess Hardwick 2024. Lifeplan Audio Recording Protocol. **protocols.io** **https://dx.doi.org/10.17504/protocols.io.kqdg3xbp1g25/v2**Version created by **Deirdre Kerdraon**

License: This is an open access protocol distributed under the terms of the **Creative Commons Attribution License**, which permits unrestricted use, distribution, and reproduction in any medium, provided the original author and source are credited

Protocol status: Working
We use this protocol and it's working

Created: April 22, 2024

Last Modified: May 08, 2024

Protocol Integer ID: 98584

Keywords: AudioMoth, audio recording, bird song, bat recording, bird recording, Lifeplan, bird sound, soundscape

Funders Acknowledgement:
European Union's Horizon 2020 research and innovation programme
Grant ID: 856506

Abstract

Here we describe a protocol that is used for collecting audio recordings of birds and bats using an AudioMoth (**AudioMoth | Open Acoustic Devices**). The protocol details the process of setting up the recording device and using it in the field. It is divided into four sections: 1) Setting up the device; 2) Site selection and placement; 3) Installation in the field and weekly sampling; and 4) Troubleshooting common problems. The protocol relies on apps and files that are free to download. The use of this protocol yields hours of recordings, mostly of birds, and can be used with developing technologies, such as Bird Sounds Global (**Bird Sounds Global web portal | WILDLABS**), to identify different species by songs and calls.

Image Attribution

Arielle Farrell

Guidelines

The aim of this protocol is to provide a standardised audio sampling method for environmental audio recordings using an AudioMoth and to include the same metadata for all samples.

This protocol will produce samples of weekly audio recordings linked to the following information:

- GPS coordinate of each AudioMoth
- Unique ID for each AudioMoth
- Coordinated universal date and time (UTC) of each recording
- Ambient temperature at the time of recording
- Country in which the sample is collected
- Name of the person that collected the sample
- Date at which the sample was placed and collected

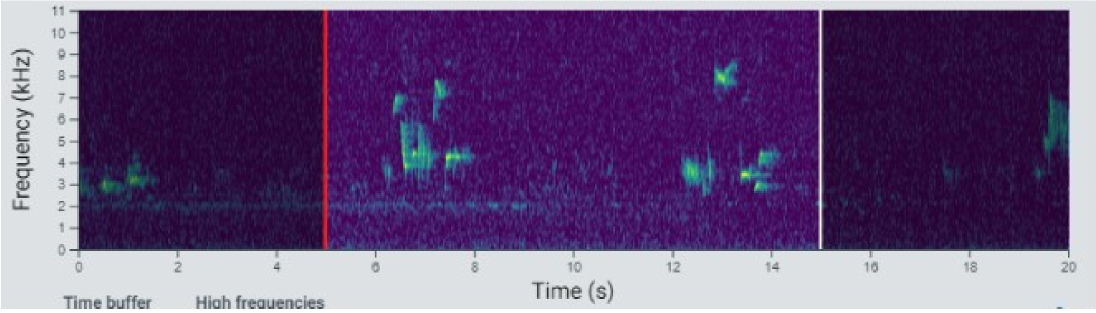

The recordings will produce spectrograms of varying length, which can be used for automated species identification

Materials

- 3 or 5 AudioMoth 1.1.0 recorders (amounts depend on site design)

Equipment	
**Audiomoth 1.1.0**	NAME
Audio recorder	TYPE
Open Acoustic Devices	BRAND
Audiomoth 1.1.0	SKU
https://www.openacousticdevices.info/audiomoth	LINK

- 3 or 5 Waterproof cases
- 3 or 5 adjustable straps
- 18 or 30 rechargeable AA batteries (such as Eneloop)
- AA battery charger
- 6 or 10 microSD cards (64 GB) with adapters.
- 2 Coloured dot stickers
- Memory card reader
- 3 external hard drives

- USB DATA cable

- Smartphone

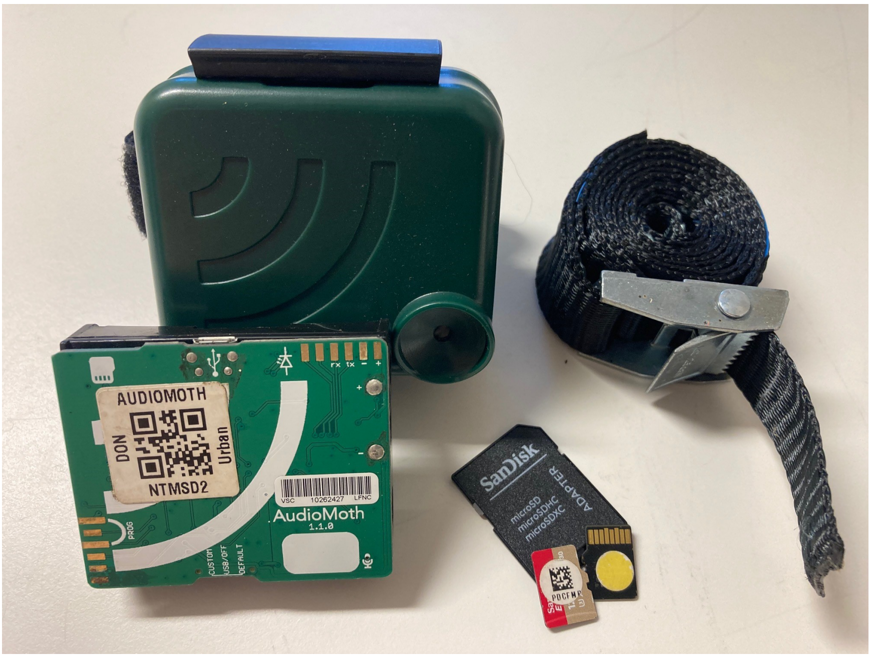

Clockwise from left: AudioMoth with Lifeplan ID code, waterproof case, strap, microSD cards (middle marked with coloured dot sticker) with adapter

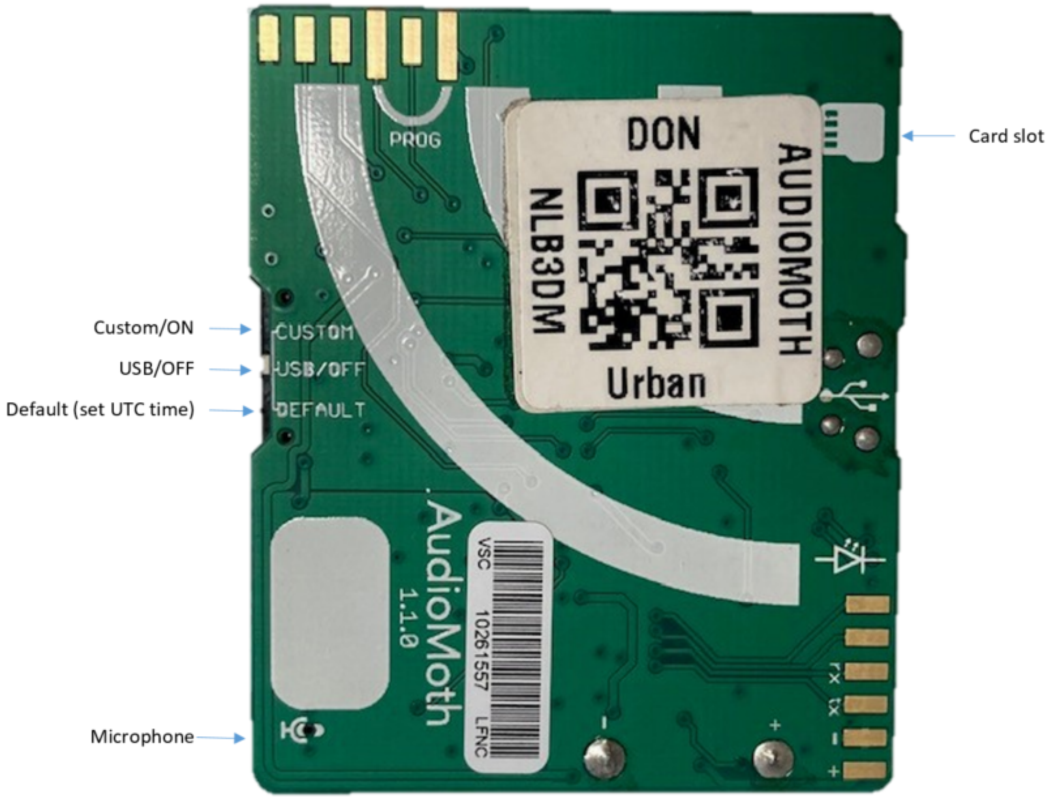

Before start

Make sure you have obtained the necessary permits for putting up AudioMoths according to local legislation and land ownership. Audio recording in public spaces may not be permitted in your country.

Device Set-up

1 **Update firmware for LIFEPLAN recording regime**

The LIFEPLAN firmware is designed for AudioMoth 1.1.X . It enables the AudioMoth to set the time in UTC using the AudioMoth chime app on a smartphone. It also encrypts all recordings for privacy protection: the encryption key will not be made available.

LIFEPLAN_Firmware_Update.pdf 100KB

- Download and install AudioMoth's Flash app on your computer: https://www.openacousticdevices.info/applications
- Download the unencrypted firmware: https://github.com/OpenAcousticDevices/AudioMoth-LIFEPLAN/releases/download/0.1.5/AudioMoth-LIFEPLAN-0.1.5.bin
- Open the Flash app on the computer, connect the AudioMoth to the computer with the USB cable
- Select "Use local file" in the flash app.
- Click on "Select file" and choose the Lifeplan firmware "*audiomoth_lifeplan_0.1.5.bin*" then click on "Flash AudioMoth"
- Once the AudioMoth is flashed, the textbox should say "*audiomoth lifeplan (0.1.5)*" is installed.
- Once the process is complete, disconnect the USB cable, then remove and reinsert batteries before use. A newly flashed AudioMoth must have its power cycled before use.
- Label your three or five AudioMoths with distinct names.
- Mark one of the AudioMoths with a coloured-dot sticker and/or write the number 5 in the white square: this AudioMoth will go in the middle of the plot.

2 **Prepare the 64 GB microSD cards**

You will need two microSD cards for each AudioMoth (6 or 10 cards, depending on the site design; see step 5).

The AudioMoths have two configurations: one for the middle AudioMoth and one for the corner AudioMoths.

2.1 The middle AudioMoth will be programmed to record continuously at 48 kHz with a record/sleep cycle of 12 hours minus 5 seconds of recording followed by 5 seconds sleep for at least the first 48 hours and then revert to recording for 1 minute out of every 10 minutes for the remainder of the deployment.

Mark two cards with a coloured dot sticker, they will be used for the middle AudioMoth.

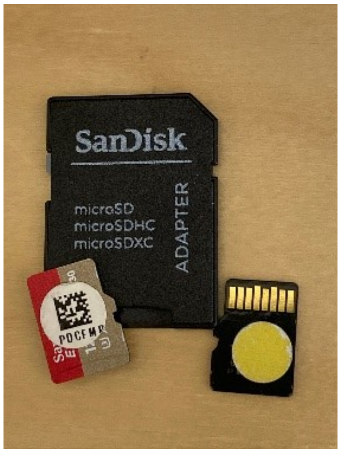

MicroSD cards with Lifeplan code sticker (left) and sticker to indicate MIDDLE (right) with adapter

- Paste the following text in a text editor and save as CONFIG.txt in a folder named "MIDDLE"

```
MIDDLE This text is ignored.

{
    enableLED: 1,
    enableBatteryLevelDisplay: 1,
    enableProprietaryFileFormat: 1,
    initialSleepRecordCycles: 4,
    initialSleepRecordCycle: {
        sleepDuration: 5,
        recordDuration: 43195
    },
    sleepRecordCycle: {
        sleepDuration: 540,
        recordDuration: 60
    },
    standardSettings: {
        gain: 2,
        sampleRate: 48000
    },
    recordingPeriods: [
        {startMinutes: 0, stopMinutes: 1440}
    ]
}

Text here is also ignored.
```

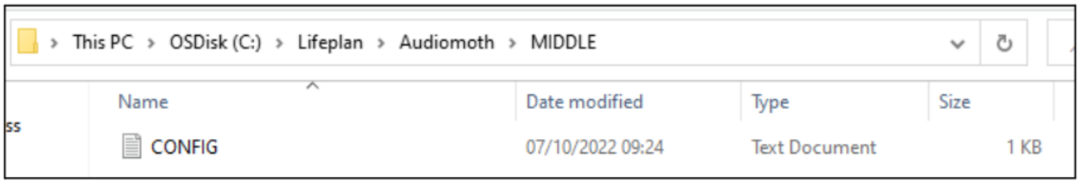

Save this CONFIG file into a folder named "MIDDLE"

- Copy the CONFIG.txt file to the two middle microSD cards.

Note

The AudioMoth will only run the program if the .txt file is named “CONFIG” and must be written in capital letters.

2.2 The corner AudioMoths will be programmed to record for 1 minute every 10 minutes at 48 kHz to record birds. They make opportunistic recordings at 384 kHz between these recordings using an amplitude threshold and high-pass filter to record bats with a daily total file size limit of 1024MB and a maximum individual file duration of 3 minutes.

- Paste the following text in a text editor and save as CONFIG.txt in a folder named "CORNER"

```
CORNER Any text here is ignored.

{
    enableLED: 1,
    enableBatteryLevelDisplay: 1,
    enableProprietaryFileFormat: 1,
    sleepRecordCycle: {
        sleepDuration: 540,
        recordDuration: 60
    },
    standardSettings: {
        gain: 2,
        sampleRate: 48000
    },
    opportunisticSettings: {
        gain: 2,
        sampleRate: 384000,
        filter: {
            lowerFrequency: 48000,
            higherFrequency: 192000
        },
        amplitudeThreshold: 512,
        maximumDuration: 180,
        maximumTotalFileSize: 1024
    },
    recordingPeriods: [
        {startMinutes: 0, stopMinutes: 1440}
    ]
}

Text here is also ignored.
```

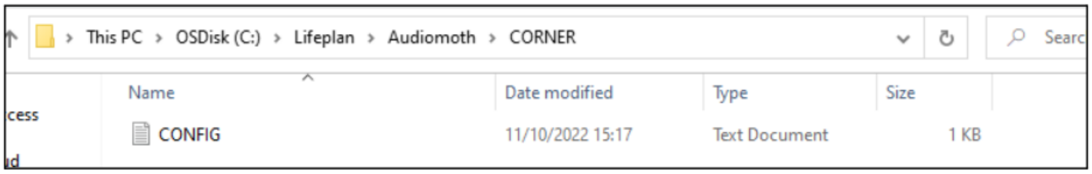

Save this CONFIG file into a folder named "CORNER"

- Copy this CONFIG.txt file to the 4 or 8 corner microSD cards.

Note

The AudioMoth will only run the program if the .txt file is named “CONFIG”. We recommend saving the two CONFIG files in separate folders to avoid confusion

3 Label each microSD card with a unique code written on the card and rename the card with the same code.

Note

Lifeplan teams use six-character unique codes printed as a QR code and placed on the microSD card. This code is scanned in conjunction with the AudioMoth QR code in the Lifeplan app to collect the metadata.

- Take the microSD cards out of their adapters
- Label the microSD cards and their respective adapters with the same code
- Keep the adapters for transporting microSD cards to and from field; the microSD cards are small and easily lost, so it is advisable to transport them in a small box

4 Install the free AudioMoth app by Open Acoustic Devices on a smartphone; it is available on App Store and Play Store. The app is used to set the correct UTC time on the AudioMoths in the field by playing a chime.

Note

See Weekly Sampling, steps 10 and 11 to set the time before you first go into the field to check that the microSD card installation worked properly.

Site Selection and Placement

5 **Site design**

The Lifeplan equipment is deployed within a 1 hectare plot. We aim for an accuracy of 10 meters.

There are two design options to choose from depending on budget and site access. In Lifeplan, we started with Option 1 in 2021, and reduced to Option 2 in 2023 by removing two of the AudioMoths.

Option 1: Four AudioMoths are set at the four corners of the 1 hectare square, with a fifth AudioMoth in the centre.
Option 2: Three AudioMoths are set up diagonally within the plot, with about 70 metres between each trap and 140 metres in a straight line between the farthest traps.

Note

If you are following the full Lifeplan sampling protocol, a camera will be on the same tree as the audio recorder. In this case, use the specifications for the camera to select a tree (see camera protocol).

Protocol

NAME

Lifeplan Camera Trapping Protocol

CREATED BY

Hanna M.K. Rogers

PREVIEW

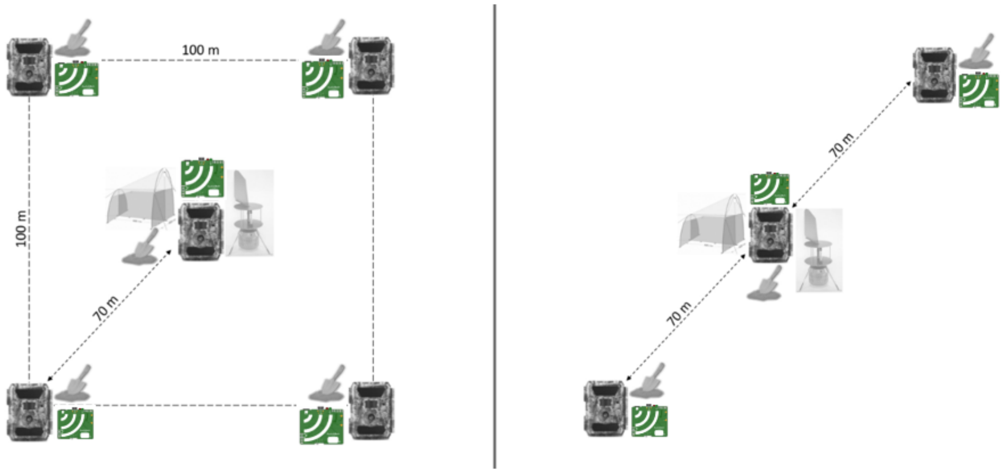

Diagrams of the two different Lifeplan plot designs, with AudioMoths shown in colour and the other Lifepland equipment shown in grey. Left: Option 1 with five AudioMoths, Right: Option 2 with three AudioMoths

6 **Install waterproof cases**

Attach the three or five waterproof cases to trees at a height between 1 and 2 meters. The case latch should face upwards if it is around a vertical tree trunk or the whole case should face downwards if it is attached to a horizontal branch to avoid rainwater penetration through the microphone membrane.

We aim for an accuracy of about 10 metres. If there are no trees, put up sturdy posts where the cases can be mounted at about 1 or 2 m height.

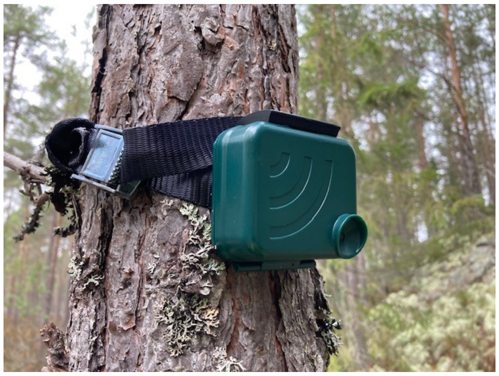

Waterproof case installed on a tree

7 Record a GPS point for each AudioMoth and associate the point with the AudioMoth ID. The GPS point will have to be linked to each collected recording.

8 Place appropriate signage by the recorders according to local legislation.

Weekly Sampling

1w

9 **Materials to bring to the sampling points:**

- Smartphone with the AudioMoth chime app
- 3 or 5 AudioMoths, depending on site design
- Enough batteries for all the AudioMoths
- Enough microSD cards (with corner or middle CONFIG.txt files)
- GPS
- Signage according to local legislation (if not already placed)

> **Note**
>
> See Site Selection and Placement, step 6 for instructions on field installation of the waterproof AudioMoth cases

10 **Sample placement**

10.1 Insert microSD cards into the AudioMoths (slot marked with picture of card)

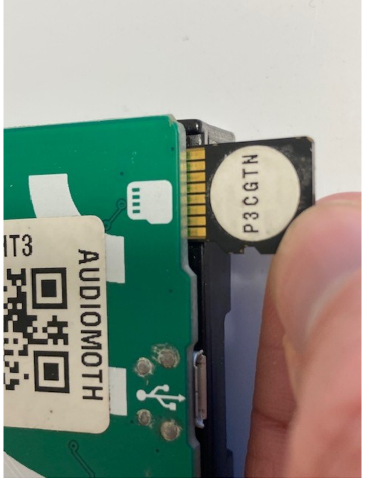

MicroSD should be positioned such that the shape matches the picture on the AudioMoth

10.2 Push in until you feel a click

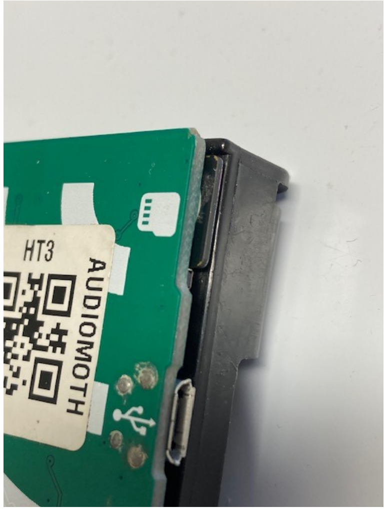

Fully inserted microSD card

10.3 Check that the switch is on USB/OFF

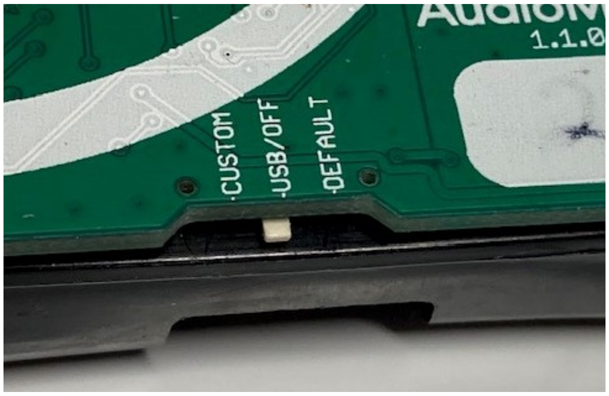

10.4 Insert three charged batteries into the AudioMoth

10.5 Take note of the date and time, AudioMoth code and microSD code associated to the AudioMoth

Note

Lifeplan teams use an app to scan a QR code on both the microSD card and AudioMoth. The app records: that it is a placement, the date and time, GPS location, microSD card code, AudioMoth code, name of collector.

11 **Set time on the AudioMoth**

Set the switch to DEFAULT. The green light will flash.

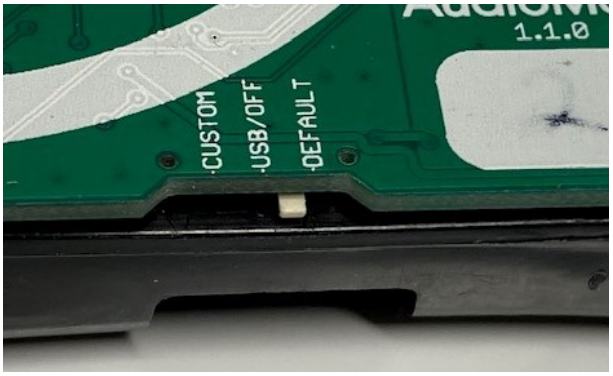

Open the AudioMoth app on your phone and play the chime. The green light should now stay on.
If the light is still flashing, increase the volume and bring the phone speaker closer to the microphone (microphone symbol on the AudioMoth).

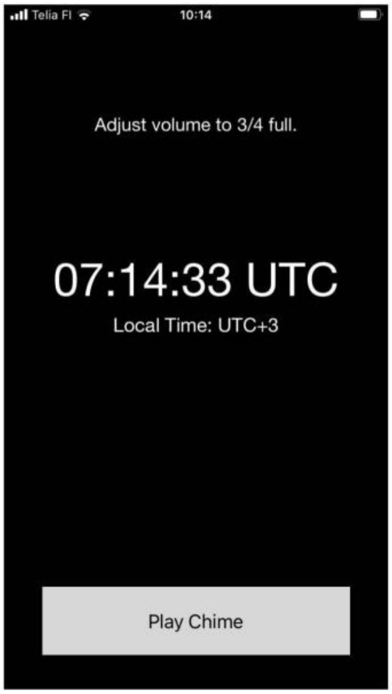

How the AudioMoth app looks on a smart phone - press the "Play Chime" button to chime the device.

12 **Start recording**

12.1 Switch to CUSTOM to start recording. Either the green or red light should begin to flash, but not both (this would indicate an error; see “Troubleshooting”).

Green light flashing = the AudioMoth is on and waiting between recordings
Red light flashing = recording

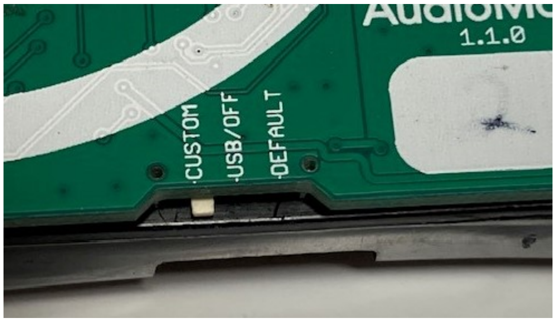

12.2 Place the AudioMoth in its waterproof case, with the microphone symbol aligned with the grey acoustic vent. Close the case.

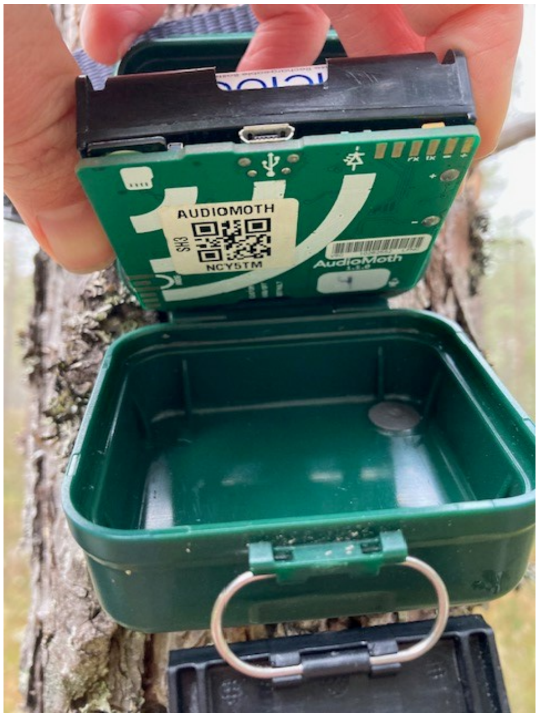

Positioning of the AudioMoth in the waterproof case; the microphone is aligned with the acoustic vent (grey membrane)

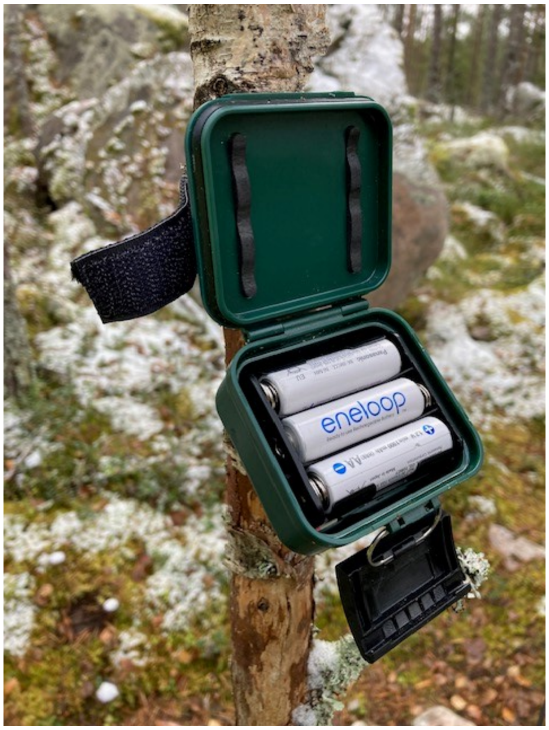

AudioMoth in waterproof case (prior to closing the case)

13 **One week later** 1w

Collect the samples on the same day each week.

13.1 Check that the AudioMoth is still working by looking at the lights: if one of the lights is flashing then the device is functioning; if both or none are flashing, an error occurred (see “Troubleshooting”).

13.2 Turn the recorder off by setting the switch to USB/OFF.

Wait for the red light to stop blinking. If a green light is on or blinking, it is not switched to USB/OFF; check the switch position and make sure it is correct.

13.3 Remove the microSD card (push on it until you hear a click, then let go and it will pop out); put the collected microSD card in its adapter (or transport box) for safekeeping.

13.4 Take note of the date and time you collected the microSD card, AudioMoth code and microSD code associated to the AudioMoth.

Note

Lifeplan teams use an app to scan a QR code on both the microSD card and AudioMoth. The app records: that it is a collection, the date and time, phone GPS location, microSD card code, AudioMoth code, name of collector.

14 Repeat steps 10-12 to place the empty microSD card for the next week of sampling.

15 **Preparation for the next sampling**

15.1 When you are back from the field, transfer the data on the microSD cards to your computer and/or external hard drive. Quickly check the folders to make sure there is a large number of audio files, indicating that no error occurred. The amount of bytes per recording should be 4146.720488 MB for a 12 hour recording and 5.760488 MB for a 1 minute recording.

15.2 Format the microSD cards before deploying them again. Remember to copy the correct CONFIG file back onto each card after formatting.

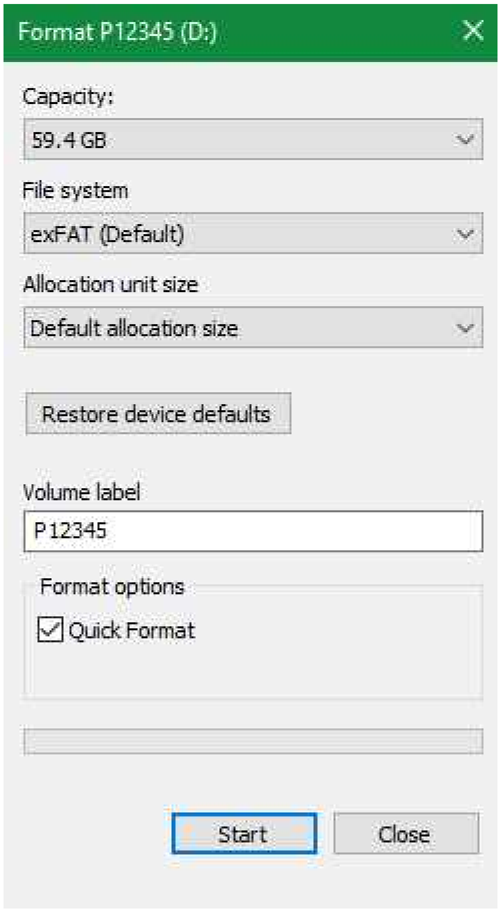

15.3 Charge batteries for the next week. Charge them as close to changing them in the field as possible to retain maximum amount of charge.

Troubleshooting

16 **There are three possible scenarios when the AudioMoth is turned on (switch is set to CUSTOM):**

1. Green light flashes once every two seconds (not recording/standby)
2. Red light flashes intermittently (recording)
3. Green and red lights flash simultaneously once every second (error)

17 **AudioMoth flashing green and red light (error), possible causes and fixes:**
Whether you are in the field or the lab/office, there are different fixes you can attempt. Try them in the following order before deciding that the AudioMoth is broken.

17.1 **The AudioMoth did not hear the chime app while in default mode.**
Try removing and replacing the batteries then switching the AudioMoth to "default" mode and playing the chime again. Once the light stays solid green, switch the AudioMoth to "custom".

> Note
>
> Make sure your phone's sound is on and set to about ¾ volume when you play the chime.

17.2 **The CONFIG file on the SD card was not renamed to "CONFIG" so the device cannot read it.**

If you are in the field, try using a different microSD card of the same variety (corner vs. middle). Make sure the card is fully inserted into the slot (you hear a click).

Make sure the CONFIG files are named "CONFIG" and that you have copied the correct one onto the microSD card after formatting.

17.3 **The SD cards have failed/glitched.**

Try doing a quick format of the card, then reinstall the CONFIG file and follow the steps above.

If you are in the field, try a different card.

17.4 **The new firmware did not get installed properly.**

On your computer, open the AudioMoth flash app and, remove all batteries and SD cards before connecting the audiomoth to the computer, check in the flash app that the firmware is called "audiomoth_lifeplan_0.1.5". Try reinstalling the firmware, then test the device by following the chime app steps.

If the AudioMoth does not function properly after this, contact the provider.

18 **Make sure to turn the AudioMoth off before removing the batteries.**

If the device is recording when the batteries are removed, the recording will be cut short, and you will see the error signal. There is a risk of corrupting the microSD card if this happens.

Always check the status of the AudioMoth options 1-3 before turning off the device to make sure it is running properly; then, after turning the switch to USB/OFF and waiting for the red light to stop flashing, remove the batteries.

19 **One LED light goes out:**

If one of the LED lights stop working, it is still possible to use the AudioMoth. Here we describe how the AudioMoth will function in either scenario.

19.1 **Green LED dies:**

The green light is relevant for chiming the AudioMoth and determining what mode it is in when set to CUSTOM.

To chime the AudioMoth without a green light, press “Play Chime” three times to better ensure that it worked.

When the AudioMoth is turned on (set to CUSTOM), if there is no light, it is on standby. If the red light is flashing intermittently, it is recording. If the red light is flashing once every second, there is an error .

19.2 **Red LED dies:**

The red light is relevant when the AudioMoth is off (set to USB/OFF) and determining what mode it is in when set to CUSTOM.

If the red light is not working when the AudioMoth is switched off, it does not make a difference in the functioning of the device.

When the AudioMoth is turned on (set to CUSTOM), if there is no light, it is recording. If the green light is flashing once every two seconds, it is on standby. If the green light is flashing once every second, there is an error .

20 **Depleted batteries**

This often happens in extreme cold temperatures, especially if the AudioMoths are left with the same batteries for two weeks, or more. In this case, the recordings will be incomplete; it will create the file but it won't actually be capable of recording and saving the sound.

You can place a thin insulation sheet in the waterproof case with the AudioMoth (on the side connected to the tree/pole, so as to not block the microphone).

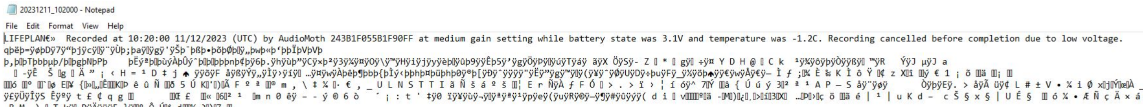

Message states that the recording was interrupted due to low voltage

21 **AudioMoth does not turn on**

If the outside temperature is around freezing, try to warm up the device (this can be as simple as putting it in a pocket close to your body for a few minutes). If it still does not turn on, remove the AudioMoth from the field. Try to turn it on again later in the office/lab; sometimes the AudioMoth will start working again after a break, especially if the outside temperatures are below freezing.

22 **AudioMoth not recognizing clock setting chime**

This can be either a software glitch or an issue with the microphone. To rule out a software glitch, try reinstalling the new LIFEPLAN firmware, then try the chime again. If that doesn't work, contact the provider to get a replacement AudioMoth.

23 **Audiomoth recorded no data**

23.1 Reinstall the firmware on the AudioMoth.

23.2 Format the microSD card (File system exFAT)

23.3 Put the correct CONFIG file on the memory card (download and see instructions in the Getting Started Guide at the top of this page)

Protocol references

Audiomoth Lifeplan Github repository: https://github.com/OpenAcousticDevices/AudioMoth-LIFEPLAN/tree/main

Background research & further reading:

Hill, A.P., Prince, P., Snaddon, J.L., Doncaster, C.P. & Rogers, A. (2019). AudioMoth: A low-cost acoustic device for monitoring biodiversity and the environment. HardwareX, 6, DOI: **10.1016/j.ohx.2019.e00073**

Hill, A.P., Prince, P., Covarrubias, E.P., Doncaster, C.P., Snaddon, J.L. & Rogers, A. (2018). AudioMoth: Evaluation of a smart open acoustic device for monitoring biodiversity and the environment. Methods in Ecology and Evolution, 9(5). DOI: **10.1111/2041-210X.12955**

Ovaskainen, O., de Camargo, U., & Somervuo, P. (2018). Animal Sound Identifier (ASI): software for automated identification of vocal animals. Ecology Letters, 21(8), 1244-1254. https://doi.org/10.1111/ele.13092
