# Supplementary material for: LIFEPLAN: A worldwide biodiversity sampling design
Source: PLoS One. 2024 Dec 31;19(12):e0313353. doi: 10.1371/journal.pone.0313353 (PMC11687782; doi:10.1371/journal.pone.0313353)
Supplement: S3 File — (PDF) [file pone.0313353.s003.pdf]

LIFEPLAN Cyclone Sampling protocol

Gaia Giedre Banelyte[1], Arielle M Farrell[1], Hanna M.K. Rogers[1], Bess Hardwick[2], Deirdre Kerdraon[1], Nerea Abrego[3]

[1]Department of Ecology, Swedish University of Agricultural Sciences;
[2]University of Helsinki;
[3]Department of Biological and Environmental Science, University of Jyväskylä

Gaia Giedre

Gaia Giedre Banelyte
Department of Ecology, Swedish University of Agricultural Sc...

ABSTRACT

Lifeplan is a global biodiversity monitoring project with the aim of assessing the current state of biodiversity worldwide, and using this knowledge to generate predictions of how biodiversity might look in the future.

In this protocol we describe the materials and method used to sample fungal spores using a wind directional reverse flow cyclone sampler within the LIFEPLAN project. This is done on a global scale and in a wide variety of environmental conditions and habitats. The aim is to identify species in further analysis (e.g. DNA sequencing) and create species lists for different locations across the globe. This protocol contains a detailed description from setup of the cyclone sampler in the field to weekly data collection, as well as steps to remove objects such as insects or other organic matter, freeze dry the samples and collect necessary metadata to help in species identification.

We identify the equipment used in Lifeplan, but also give technical specifications of that equipment so that other users of this protocol can find equivalent alternative equipment. We also specify what metadata should be collected with the cyclone sample data. The technical solution we use to collect metadata in the Lifeplan project is described in detail in the full Lifeplan protocol.

It is critical that we employ standardized operating procedures for the cyclone sampling. Our coordinated efforts will ensure specimen preservation for sequence analysis and high data quality, permitting the comparison of sites at a global scale. For global standardization with the BIOSCAN initiative, of which LIFEPLAN is a part. The species identification process is outside the scope of this protocol and will be described elsewhere.

Protocol Info: Gaia Giedre Banelyte, Arielle M Farrell, Hanna M.K. Rogers, Bess Hardwick, Deirdre Kerdraon, Nerea Abrego . LIFEPLAN Cyclone Sampling protocol. **protocols.io** https://protocols.io/view/lifeplan-cyclone-sampling-protocol-c7twznpe

Created: Jan 19, 2024

Last Modified: Mar 01, 2024

PROTOCOL integer ID: 93782

Keywords: metabarcoding, fungi, lifeplan, biodiversity, cyclone sampler

Funders Acknowledgement:
European Union's Horizon 2020 research and innovation programme
Grant ID: 856506

MATERIALS

Materials needed for the whole process:

- Cyclone sampler adapted for screw-top lid tubes

Equipment	
**Cyclone sampler for field operation**	NAME
MYCOLOGICAL AND ENTOMOLOGICAL INSTRUMENTS	TYPE
Burkard Manufacturing	BRAND
Cyclone sampler for field operation	SKU

- 12V car batteries or equivalent
- Sterile screw top 1.5 ml microtubes with O-ring lid
- 70 % ethanol
- Bactericide and fungicide cleaning agent such as: Virkon® , 1 % chlorine or Trigene®
- Sterile dry wipes
- disposable gloves for laboratory use
- Cardboard microtube boxes
- Plastic microtube boxes
- MARS Lateral flow fume hood
- Aluminium foil
- Autoclave
- Sterile tweezers
- Millipore Milli-Q lab water system or equivalent
- 250 ml sterile reusable glass bottles
- Pipette 1000 µL
- Pipette tips 1000 µL
- Pop-lid 1,5 or 2 ml tubes
- Microtube rack
- Single tube vortex mixer
- Permanent marker (size Small)
- Paper for labelling
- Tape
- Autoclavable disposal bags for tubes and pipette tips
- Empty container for used tweezers (e.g. plastic beaker)
- Freeze dryer

- Optional: timer

BEFORE START INSTRUCTIONS

Ensure that all proper specimen permissions are obtained (i.e. from local authorities, property owners, etc.). Consider possibilities of wildlife disturbance and/or human vandalism; the trap may be relocated if consistent issues persist after deployment.

Site selection and installation of the cyclone sampler

1 **Site design**

The Lifeplan equipment is deployed within a 1 hectare sampling plot.
There are two design options which are to be chosen depending on budget and site access possibilities. In the first option, five audio recorders and cameras are placed within the same plot, whereas in the second option the cyclone sampler is accompanied by three audio recorders and cameras. In both designs, the cyclone sampler is placed within the 1 ha square as close to the center as possible.

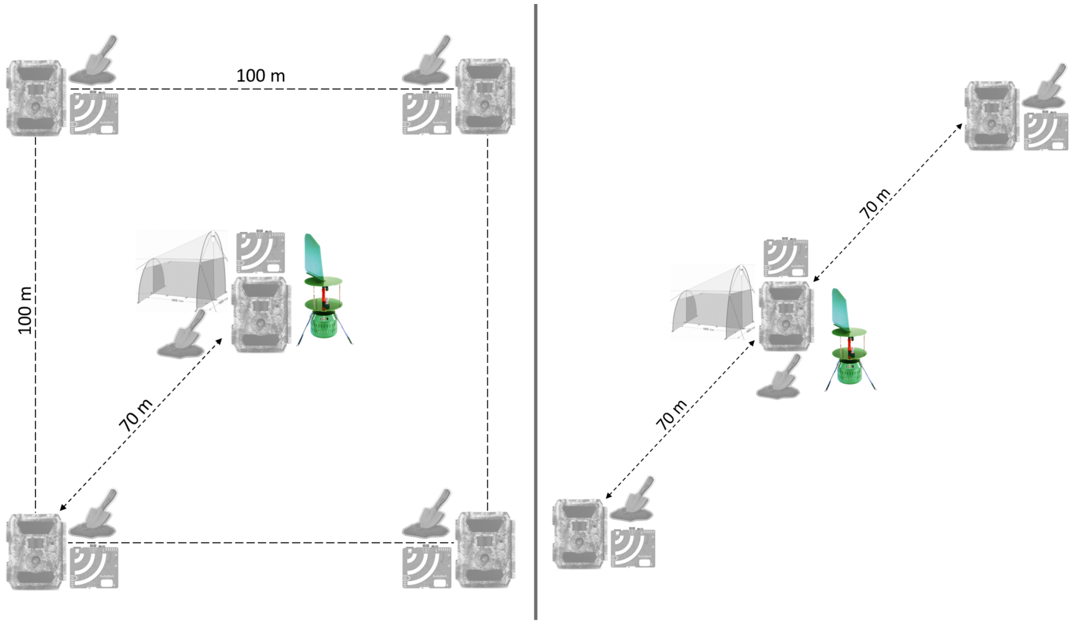

Diagrams of the two different Lifeplan plot designs, with a cyclone sampler shown in colour and the rest of the Lifeplan equipment shown in grey. Left: Option 1 with five audio recorders and cameras, Right: Option 2 with three audio recorders and cameras

2 **Site selection**

Select a site that:

- Is roughly in the center of the selected one-hectare plot.
- Is easy to access, since you might need to carry heavy batteries. To avoid dust from traffic in the samples, it is recommended that the sampler is at least 15 meters from roads.
- Has room for the sampler's wind plate to rotate freely.
- Has room for the battery to stay next to it, and it is possible to protect the battery from rain or alternatively the cyclone sampler has access to a power supply. If possible, it is recommended to construct a roof of ca. 2 m^2 to cover the cyclone sampler and protect it from rain (while not disrupting the spore sampling).
- Has flat, firm ground. When placing the cyclone sampler ensure that is level with a spirit level, and that the legs of the device do not sink in the ground. This will enable the wind plate to rotate and direct the sampler according to the wind direction.

Bi-weekly sampling

3 **Sampling frequency:**
Collect two 24 h samples twice a week during the sampling season.
Depending on your location and climate, the sampling period can cover the whole year-round or only the frost-free period.
Aim to collect the samples on the same two days each week.

> **Note**
>
> In LIFEPLAN, one weekly sample is sent for metabarcoding, the other one is archived in -20 °C freezer.

3.1 If you have a long travel time to the sampling plot and need to reduce the number of weekly visits, you can:

1. Set up a timer which cuts off power to the sampler after 24 hours. In this way, you can save a third visit and collect the tube on the following sampling week.

> **Note**
>
> The cyclone sampler will keep running until the power source runs out so it is important to take precautions to ensure the sampling time lasts 24 h.
>
> Ensure that the timer works correctly before using it in the field.

2. Only collect one sample per week (in which case there will be no archived samples).

4 The sampler comes with crocodile grips for connecting it to a 12 V car battery:

4.1 Connect the car battery or other power supply to the cyclone sampler.

4.2 Check that the cyclone sampler runs smoothly by listening to the sound of the motor when switching the power on.

4.3 The battery should have enough power to run a minimum of 24 hours and needs to be recharged as required

5 **Sample placement:**

Put on disposable gloves. Label your vial clearly with the **sample location, placement date and time** and other relevant information. You may write on a small piece of paper and tape it onto the vial.

Note

For the LIFEPLAN project, this information is collected by scanning a QR code in the LIFEPLAN App.

6 Remove the cyclone unit:
A grey plastic release knob is located under the lower plate. Hold the cyclone sampler unit with one hand while while pulling the release knob downwards (1). Tilt the cyclone unit to remove it (2).

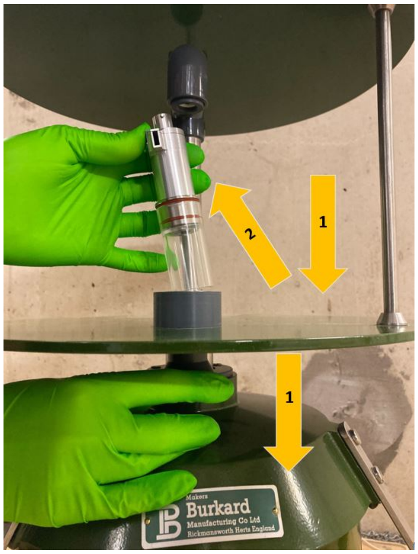

7 Unscrew the lid of your prepared vial and store the lid in a clean container for closing the vial after sampling. Using the grey tool, fit the empty vial into the cyclone unit. Place the cyclone unit back to its place.

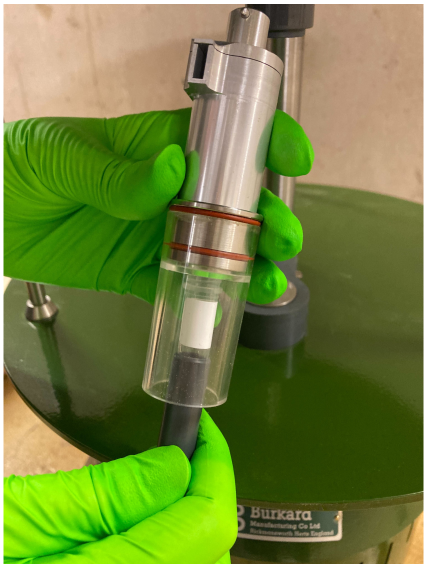

8 **Sample collection:**

8.1 After 24 hours, disconnect or switch off the power source from the sampler.

Note

If using a timer, regularly check the status of the power source.

8.2 Follow go to step #6 and go to step #7 to remove the vial from the cyclone sampler. When closing the lid, avoid touching the inner part.
Note the **collection date and time, the duration of sampling** and other relevant information. Place the vial in a clean storage box or bag. Store in a -20 °C freezer as soon as possible.

Note

For the LIFEPLAN project, the metadata is collected by scanning a QR code in the LIFEPLAN App.

Processing of samples in the laboratory

9 **Preparations before cleaning samples**

9.1 **Sterilizing tweezers:**

- Soak all dirty tweezers in a cleaning solution (such as chlorine, Virkon® or Trigene®) for at least 15 minutes.
- Rinse thoroughly with deionized water while rubbing the tips with gloved hands to remove any insect residue.
- Wipe each tweezer dry with sterile paper towels, such as Kimtech.
- Wrap batches of tweezers in two layers of aluminium foil.
- Autoclave at 121 °C for 30 minutes.
- Optional: if your fume hood has UV light, clean the surface, open foil containing tweezers and place under UV light for 30 minutes before use.

9.2 **Clean the plastic microtube boxes:**

- Wipe box first with the cleaning agent, leave for 10 minutes, and then 70 % ethanol, using sterile dry wipes.
- Place the empty plastic microtube box under UV light for 30 min.

10 **Prepare workspace:**

10.1 Take a box of vials out of the freezer for defrosting (approx. 30 min to 1 hr).

10.2
- Put on disposable gloves. Clean fume hood surface: First with cleaning agent, wait 10 minutes, wipe with sterile wipes Then spray the surface with 70 % ethanol and leave to dry.

10.3 Set up the bench in the fume hood:
- bottle of MilliQ water
- pop-lid microtubes
- microtube rack
- sterile tweezers
- empty container for used tweezers
- pipette
- box of pipette tips
- autoclavable disposal bag
- permanent marker
- clean plastic microtube box
- single tube vortex mixer

10.4 Set up approximately 10 open pop-lid tubes in the microtube rack and fill each tube with 500 µL of MilliQ water using the pipette; dispose of the pipette tip.

10.5 Turn on UV light and leave for approximately 30 minutes. Optional: open the foil of the tweezers while under the UV light.

Note

Important: Do not have samples in or near the fume hood while the UV light is on.

11 **Cleaning:**

11.1 Put on new disposable gloves. Carefully open a sample and check if there are any insects or insect parts (in the lid as well), check for soil/dust.

Note

Make sure not to touch the inside of the lid or the rim of the tube.

11.2 **If the sample does not have an insect, or large piece of organic matter (1mm+)**: Close the vial and place it in the plastic microtube box. Mark soil/dust, water, etc. on the lid, if needed. If there is more than 25 µL of soil, add a mark on the label as well.

If the sample has an insect, or large piece of organic matter (1mm+): Add 500 µL MilliQ water, close the lid, and vortex the sample (on speed 5 for a few seconds). This way the fungal spores attached to the insects come loose and stay in the sample.

If the sample contains water, you only need to mark the vial with the water symbol.

Insect	Water	Dust	More than 25 µL of soil/dust	Insect + Water	Insect + Dust
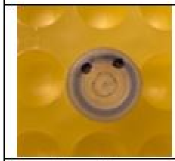	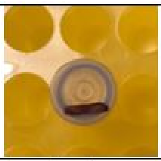	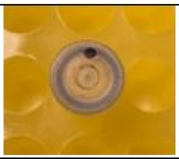	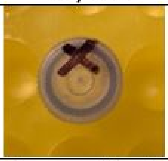	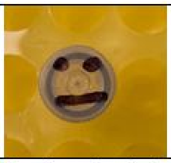	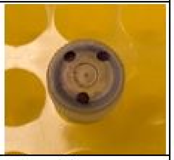
Two dots	One line	A single dot	A cross on lid and label	One line + two dots	Two dots on one side, single dot opposite

Symbols for indicating different issues with the spore samples.

11.3 Remove the insect/organic piece with tweezers and place it in a pop-lid tube. If there still are insect parts/organic pieces in the sample, rinse the tweezer in the pop-lid tube and remove all of the particles. You can use the same tweezer and the same discard pop-lid tube for the same cyclone vial.

11.4 Close and discard the tube with the insect/organic matter in it.

11.5 Place the used tweezer in the container for used tweezers.

11.6 Close the sample and mark with the relevant symbol(s) on the lid go to step #11.2 .

11.7 Place the cleaned sample in the plastic microtube box.

11.8 Repeat the process using a new pair of tweezers and a new pop-lid tube for each sample. If you have more than one sampling site, change gloves between sites.

11.9 Store the samples in a -20 °C freezer.

Freeze drying cyclone samples

12 **Preparations before freeze drying:**

12.1 Clean bench surfaces: first with cleaning agent, leave for 10 minutes, dry with sterile wipes, and then 70% ethanol, dry with sterile dry wipes.

12.2 Unscrew the lids slightly so moisture can escape, but not loose enough to fall off.

12.3 Close the microtube box and place in freezer for at least 30 minutes, until all water is frozen. If the water is still liquid, leave it in the freezer for another 30 minutes.

13 Freeze dry the samples until there is no visible moisture remaining. For the LIFEPLAN project, we used a COOLSAFE 55-4 freeze dryer (Labogene) at -50 °C for at least 48 hours.

14 **After freeze drying:**

14.1 Tighten lids on sample vials.

14.2 Return the samples to a -20 °C freezer for storage until ready for further analyses.
