# Supplementary material for: LIFEPLAN: A worldwide biodiversity sampling design
Source: PLoS One. 2024 Dec 31;19(12):e0313353. doi: 10.1371/journal.pone.0313353 (PMC11687782; doi:10.1371/journal.pone.0313353)
Supplement: S4 File — (PDF) [file pone.0313353.s004.pdf]

Dec 11, 2023

Global Malaise Trap Project and LIFEPLAN Malaise sampling V.2

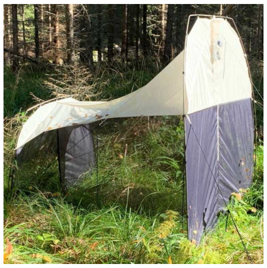

DOI
dx.doi.org/10.17504/protocols.io.kqdg3xkdqg25/v2

Gaia Giedre Banelyte[1], Arielle M Farrell[1], Hanna M.K. Rogers[1], Bess Hardwick[2], Deirdre Kerdraon[1], Kate HJ Perez[3], Jeremy R. deWaard[3], Jayme E Sones[4]

[1]Department of Ecology, Swedish University of Agricultural Sciences; [2]University of Helsinki;
[3]Centre for Biodiversity Genomics, University of Guelph; [4]Centre for Biodiversity Genomics

Gaia Giedre Banelyte
Department of Ecology, Swedish University of Agricultural Sc...

OPEN ACCESS

DOI: dx.doi.org/10.17504/protocols.io.kqdg3xkdqg25/v2

Protocol Citation: Gaia Giedre Banelyte, Arielle M Farrell, Hanna M.K. Rogers, Bess Hardwick, Deirdre Kerdraon, Kate HJ Perez, Jeremy R. deWaard, Jayme E Sones 2023. Global Malaise Trap Project and LIFEPLAN Malaise sampling. **protocols.io** https://dx.doi.org/10.17504/protocols.io.kqdg3xkdqg25/v2Version created by Deirdre Kerdraon

License: This is an open access protocol distributed under the terms of the **Creative Commons Attribution License**, which permits unrestricted use, distribution, and reproduction in any medium, provided the original author and source are credited

Created: December 11, 2023

Last Modified: December 11, 2023

Protocol Integer ID: 92104

Keywords: Malaise, invertebrates, lifeplan, bioscan

Funders Acknowledgement:
European Union’s Horizon 2020 research and innovation programme
Grant ID: 856506
Government of Canada's New Frontiers in Research Fund
Grant ID: NFRFT-2020-00073
Government of Canada's Large Scale Applied Research Program administered by Genome Canada and Ontario Genomics
Grant ID: OGI-208
Government of Canada's Major Science Initiatives Fund administered by Canada Foundation for Innovation
Grant ID: 42450

Abstract

Lifeplan is a global biodiversity monitoring project with the aim of assessing the current state of biodiversity worldwide, and using this knowledge to generate predictions of how biodiversity might look in the future.

In this protocol we describe the materials and method used to sample flying insects with a Malaise trap for the Global Malaise Trap Project and the LIFEPLAN project on a global scale and in a wide variety of environmental conditions and habitats. The aim is to identify species in further analysis (e.g. image recognition, DNA sequencing) and create species lists for different locations across the globe. This protocol contains a detailed description from setup of the Malaise traps in the field to weekly data collection, as well as steps to reduce ethanol in the samples and collect necessary information to help in species identification.

We identify the equipment used in Lifeplan, but also give technical specifications of that equipment so that other users of this protocol can find equivalent alternative equipment. We also specify what metadata should be collected with the Malaise trap data. The technical solution we use to collect metadata in the Lifeplan project is described in detail in the full Lifeplan protocol.

It is critical that we employ standardized operating procedures for the Malaise trapping. Our coordinated efforts will ensure specimen preservation for sequence analysis and high data quality, permitting the comparison of sites at a global scale. For global standardization with the **BIOSCAN** initiative, of which LIFEPLAN is a part. LIFEPLAN is based on bulk processing (metabarcoding) of samples and automatic image recognition which are outside of the scope of this protocol and will be described elsewhere.

Guidelines

This protocol is used by the BIOSCAN initiative for the Global Malaise Program and the LIFEPLAN project

The aim of this protocol is to provide a standardised Malaise trap sampling method to collect invertebrates using a Malaise trap across the world and include the same metadata for all samples.

This protocol will produce samples of weekly invertebrate catches conserved in pure ethanol linked to the following information:

- Unique sample ID
- GPS coordinate of the Malaise trap
- Country in which the sample is collected
- Name of the person that collected the sample
- Date at which the sample was placed
- Date at which the sample was collected

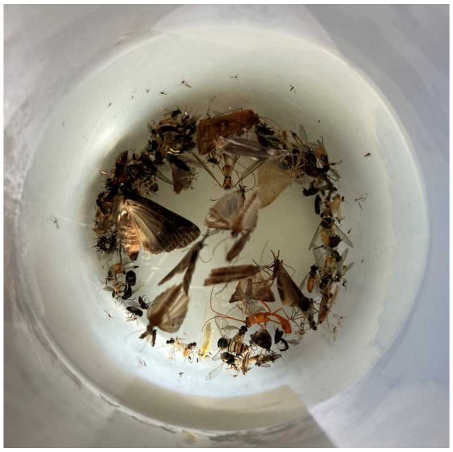

Example of a malaise sample

For the LIFEPLAN project, the metadata is automatically collected with the use of the LIFEPLAN app. A QR code linked to a unique 6 character code for each sample is scanned when the sample is placed, then scanned again when the sample is collected. For the Lifeplan project, the Malaise trap is placed close to the centre point or middle of the LIFEPLAN sampling site.

Materials

Sampling materials:

- 1x Malaise trap kit including assembly instruction sheet

As our standard Malaise Traps, we use the model “ez-Malaise Trap” manufactured by MegaView Science Co. (Bugdorm). Compared to the commercially available design, we have made a few modifications: we use Nalgene bottles and barbed tent pegs, and we have removed the "moth excluder device" (a triangular piece of cloth with small holes, used to keep out bigger insects when focusing on Diptera and Hymenoptera).

- Plastic 500 ml Nalgene bottles
- External and internal sampling labels
- 95 % pure ethanol
- Paper tissues
- Gloves

Laboratory materials for decanting:

- 50 micron nitex filters cut into approx. 10 x 10 cm squares
- Connecting ring or rubber band
- Sterilizing and disinfecting solution (e.g. bleach, Virkon, etc.)
- Squeeze bottle with 95 % ethanol
- Ethanol waste container (e.g. glass beaker of at least 500 ml)
- Sterile wipes (e.g. Kimtech)
- Gloves
- -20 °C freezer

Malaise trap specifications:

Dimensions: L165 x W180 x H180 cm
Net Weight: 2,300 grams
Main Material: Knitted Polyester Mesh Fabric
Frame: Shock-corded Aluminium Poles
Mesh: White: 108 x 32 | 470 µm Aperture; Black: 96 x 26 | 680 µm Aperture

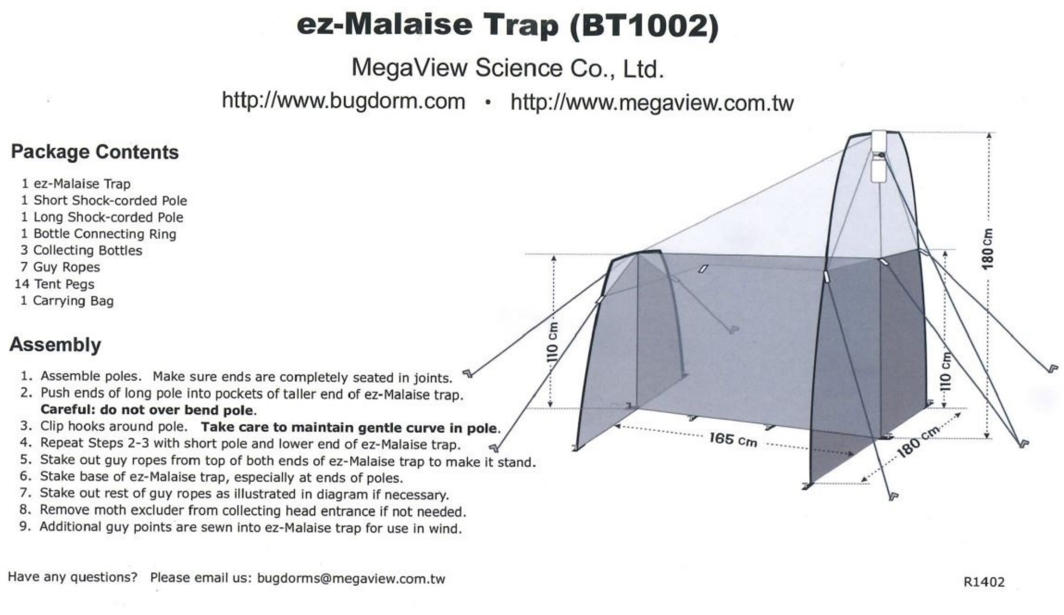

ez-Malaise Trap (BT1002)

MegaView Science Co., Ltd.

http://www.bugdorm.com • http://www.megaview.com.tw

Package Contents

1 ez-Malaise Trap
1 Short Shock-corded Pole
1 Long Shock-corded Pole
1 Bottle Connecting Ring
3 Collecting Bottles
7 Guy Ropes
14 Tent Pegs
1 Carrying Bag

Assembly

1. Assemble poles. Make sure ends are completely seated in joints.
2. Push ends of long pole into pockets of taller end of ez-Malaise trap. **Careful: do not over bend pole.**
3. Clip hooks around pole. **Take care to maintain gentle curve in pole.**
4. Repeat Steps 2-3 with short pole and lower end of ez-Malaise trap.
5. Stake out guy ropes from top of both ends of ez-Malaise trap to make it stand.
6. Stake base of ez-Malaise trap, especially at ends of poles.
7. Stake out rest of guy ropes as illustrated in diagram if necessary.
8. Remove moth excluder from collecting head entrance if not needed.
9. Additional guy points are sewn into ez-Malaise trap for use in wind.

Have any questions? Please email us: bugdorms@megaview.com.tw

R1402

Before start

Ensure that all proper specimen permissions are obtained (i.e. from local authorities, property owners, etc.). Consider possibilities of wildlife disturbance and/or human vandalism; the trap may be relocated if consistent issues persist after deployment.

Placement and deployment of the Malaise trap

1 The trap design depends on insects moving towards the highest and lightest part of the trap. When mounting, make sure that the collecting part of the trap (i.e., the trap head) is directed towards the equator (north in the Southern hemisphere and south in the Northern hemisphere). Also, check that the collecting part is the highest part of the trap, and avoid placing the trap on a downhill slope with the collecting part lowest. When possible, position the trap in areas with low undergrowth; forest edges or clearings and elevated sites are recommended.

2 At your field site, assemble the trap securely, according to the Malaise trap instruction sheet. When possible, tie the front and/or back ropes to nearby trees for added support. If your plot does not have trees or there is a risk of high winds, attach the trap poles to a 1.5 to 2 meter stake or post at its highest points to protect the trap against falling over from high winds.

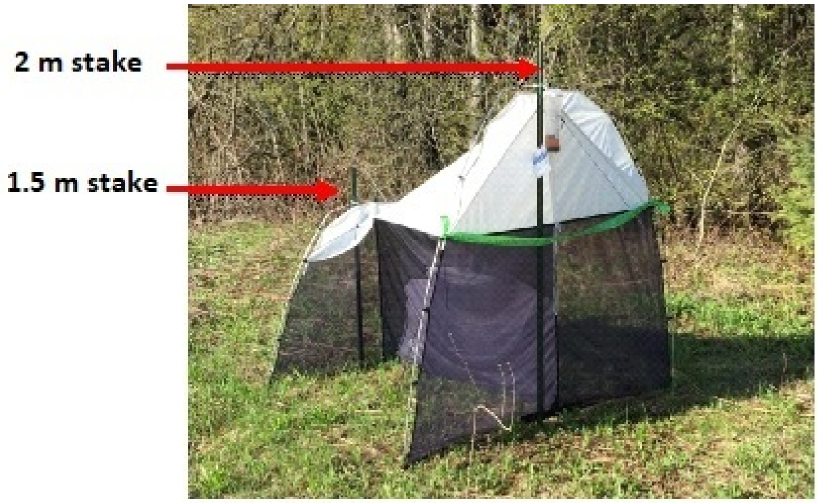

Malaise trap sampling

3 **Sampling frequency:**
Collect the sample once a week during the insect activity period (i.e., during weeks with an average temperature above freezing; occasional frosts are not an issue). Remove the sample on the same day each week.

4 Label your bottle clearly with the **sample location, placement date** and other relevant information. Be aware that ethanol erases most markers; to avoid that you can use a pencil.

Note: For the LIFEPLAN project, this information is collected by scanning a QR code in the LIFEPLAN App.

5 Fill approximately 2/3 of a sampling bottle with 95% ethanol in the laboratory, close the lid tightly.

6 Tightly affix the prepared collection bottle to the trap head; cover the bottle with foil to prevent the
UV light from damaging the DNA, tie the white ropes on the trap around the bottle to secure it. Do not cover the top bottle with foil, light needs to shine into the trap for it to work.

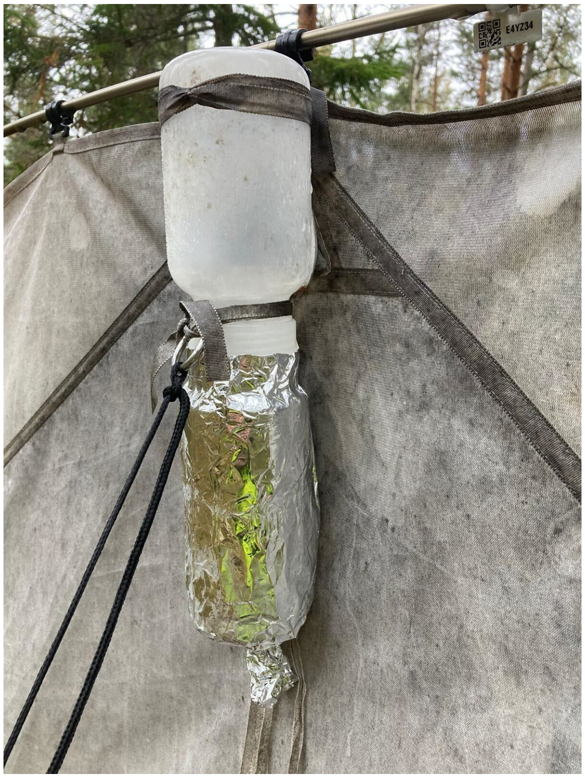

7 To collect a sample, untie the bottle and remove the foil. **Note the sample code and collection date**. You can also write on a blank unlined paper using a pencil and put that paper inside the bottle. Close the bottle tightly with the lid. If possible, store the collected sample in a cooling container in the field.

Note

Note: For the LIFEPLAN project, this information is collected by scanning a QR code in the LIFEPLAN App.

8 Before installing a new bottle, wipe the trap head with a clean paper wipe while wearing gloves to avoid dead insects remaining between weeks.

9 Repeat from step 4 to place the next sampling bottle.

10 Once in the lab, ensure that entire insect mass is fully submerged in ethanol before storage; add fresh ethanol to the sample bottle as needed. Store in a -20 °C freezer as soon as possible.

Decanting ethanol in the laboratory for transferring samples to smaller containers or shipping

11 Put on single use gloves. Clean the bench surface with sterilizing liquid and sterile wipes, then wipe once again with 70% ethanol and leave to dry.

12 Clean the nitex filter and the connecting ring with ethanol. Use one filter per Malaise trap or site.

13 Place a nitex filter on the bottle, leave a gap for air at the top. Secure with a connecting ring or a rubber band.

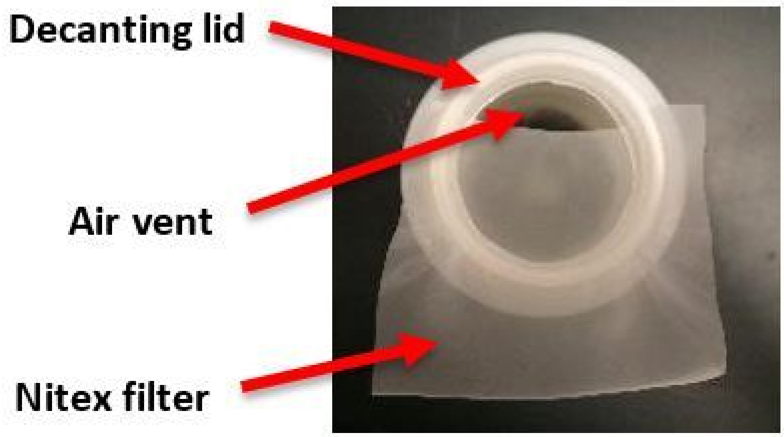

14 Decant off most of the ethanol from each bottle into the container for waste ethanol.

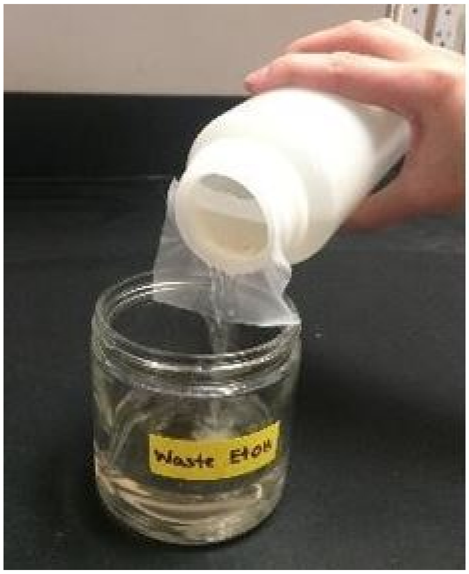

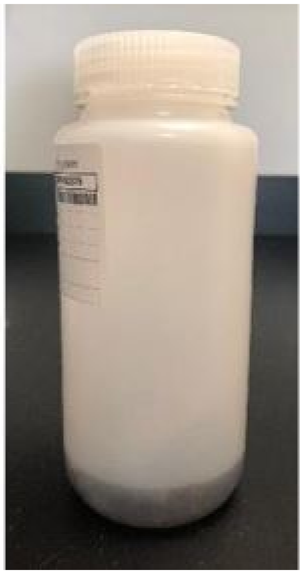

15 Carefully remove the connecting ring. Turn the nitex filter around and wash the sample contents using a squeeze bottle containing 95% ethanol back into the sample bottle. Make sure that the amount of ethanol in the sample does not exceed the desired amount.

16 Immediately store the sample in a -20°C freezer until ready for analysis or shipment.

Protocol references

Malaise, R. (1937) A new insect trap. *Entomologisk Tidskrift*, 58, 148–160.
Townes, H.K. (1972) A light-weight Malaise trap. *Entomological News*, 83, 239–247.
