# Supplementary material for: LIFEPLAN: A worldwide biodiversity sampling design
Source: PLoS One. 2024 Dec 31;19(12):e0313353. doi: 10.1371/journal.pone.0313353 (PMC11687782; doi:10.1371/journal.pone.0313353)
Supplement: S5 File — (PDF) [file pone.0313353.s005.pdf]

Feb 09, 2024 Version 2

LIFEPLAN soil sampling V.2

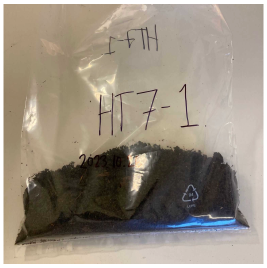

DOI
dx.doi.org/10.17504/protocols.io.5jyl8pw9rg2w/v2

Gaia Giedre Banelyte[1], Arielle M Farrell[1], Hanna M.K. Rogers[1], Bess Hardwick[2], Deirdre Kerdraon[1]

[1]Department of Ecology, Swedish University of Agricultural Sciences; [2]University of Helsinki

Deirdre Kerdraon
Department of Ecology, Swedish University of Agricultural Sc...

OPEN ACCESS

DOI: dx.doi.org/10.17504/protocols.io.5jyl8pw9rg2w/v2

Protocol Citation: Gaia Giedre Banelyte, Arielle M Farrell, Hanna M.K. Rogers, Bess Hardwick, Deirdre Kerdraon 2024. LIFEPLAN soil sampling. **protocols.io** https://dx.doi.org/10.17504/protocols.io.5jyl8pw9rg2w/v2Version created by **Deirdre Kerdraon**

License: This is an open access protocol distributed under the terms of the **Creative Commons Attribution License**, which permits unrestricted use, distribution, and reproduction in any medium, provided the original author and source are credited

Protocol status: Working
We use this protocol and it's working

Created: February 05, 2024

Last Modified: February 09, 2024

Protocol Integer ID: 94675

Funders Acknowledgement:
European Union's Horizon 2020 research and innovation programme
Grant ID: 856506

Abstract

Lifeplan is a global biodiversity monitoring project with the aim of assessing the current state of biodiversity worldwide, and using this knowledge to generate predictions of how biodiversity might look in the future.

In this protocol, we describe the method used within the Lifeplan project for collecting soil samples on a global scale in a wide variety of environmental conditions and habitats. The aim is to use this data to identify species of fungi and create species lists for the different locations across the globe. This protocol contains a detailed description on the materials needed to take soil samples, the steps for preparing the samples for further analysis (e.g. DNA sequencing), as well as necessary information to help in species identification.

We identify the equipment used in Lifeplan, but also give technical specifications of that equipment so that other users of this protocol can find equivalent alternative equipment. We also specify which metadata should be collected along with the soil samples. The technical solution we use to collect metadata in the Lifeplan project is described in detail in the full Lifeplan protocol.

The expected outcome of this method is soil samples with associated metadata. The soil fungal species identification process in LIFEPLAN is based on metabarcoding and is outside the scope of this protocol. DNA sequencing and species identification will be described elsewhere.

Guidelines

This protocol describes soil sampling for the LIFEPLAN project for standardised data collection.

The result of this protocol will be a soil sample of approximately 50 g linked to the following metadata:

- Unique sample ID
- GPS coordinates of the location of the soil sample
- Country in which the sample was taken
- Date at which the sample was taken
- Corner ID to match soil sample point to the other LIFEPLAN data

Sampling Schedule:

At each site, soil samples are taken four times a year. The four sampling events should be distributed evenly over the entire plant growing season. Thus, in the Arctic region, the sampling times will be distributed along the few months of plant growth (i.e. the freeze- and snow-free season), whereas in tropical regions the sampling events will be distributed over the whole year.

For the LIFEPLAN project, the metadata is automatically collected through the LIFEPLAN app. A QR code, linked to a unique 6 character code for each sample, is scanned when the sample is collected.

For the Lifeplan project, a soil sample combining three replicates is collected around each of the trees to which a camera trap is attached, so that three or five composite soil samples are collected in one plot at each sampling event.

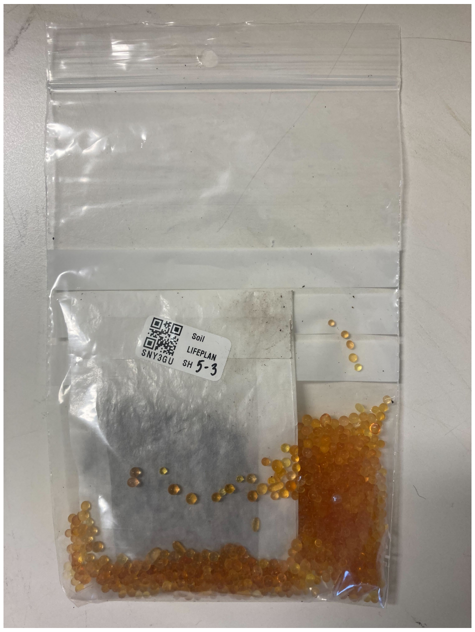

Example of a soil sample with a label containing a sample ID and corner number, inside a plastic bag with dry silica beads.

Materials

Sampling materials:

- Disposable rubber gloves
- Clear clean plastic bags for mixing soil samples
- Paper glassine bags
- Sample ID stickers / pencil
- Airtight plastic resealable bags
- Silica gel
- Gardening spade **OR** 5 cm corer **OR** knife (sharp and strong, at least 5 cm blade) and tablespoon
- Tablespoon
- Ruler to measure 5 cm depth
- 95% pure ethanol / flame
- Paper tissues
- Cooler box
- Freezer (preferred) or refrigerator for sample storage

Before start

Ensure that all proper specimen permissions are obtained (i.e. from local authorities, property owners, etc.). Consider possibilities of wildlife disturbance and/or human vandalism.

Site Selection

1 **Site design**

The Lifeplan sampling takes place within a 1 hectare plot. We aim for an accuracy of 10 m for the selection of each point. There are two design options to choose from depending on budget and site access. In Lifeplan, we started with Option 1 in 2021, and reduced to Option 2 in 2023 by removing two of the corners.
Option 1: Four points (e.g. trees) are set at the four corners of the 1 hectare square, with a fifth point in the centre.
Option 2: Three points are selected diagonally within the plot, with about 70 metres between each point and 140 metres between the farthest points.

In Lifeplan the corner/outer points are given individual numbers from 1-4, whereas 5 is assigned to the centre.

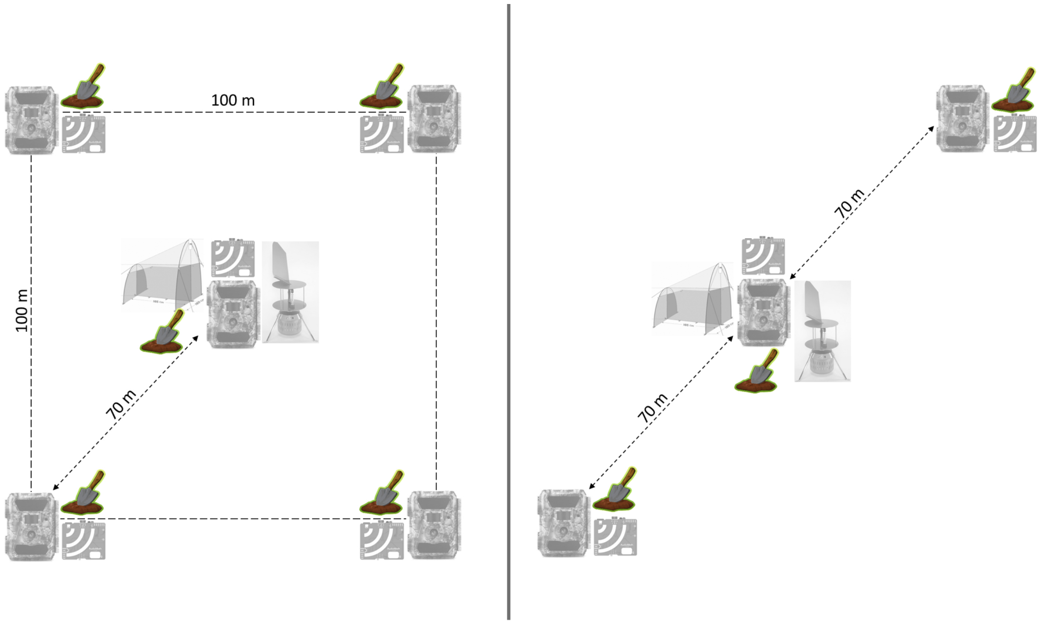

Left: option 1 with a five point design. Right: option 2 with a three point design.

2 The soil samples should be collected within a 5 m radius around the tree or post on which the LIFEPLAN camera and audio recorders have been installed.

Soil sampling

3 Prepare the sampling materials: put on rubber gloves and note the sample ID and the sampling date on the clear plastic bag with a permanent marker.

4 Squirt some ethanol on the spade, spoon, or knife and thoroughly clean it with a paper tissue. Alternatively, use a flame.

5 Remove any layer of live cryptogams (including moss, lichen or algae) and loose debris from the sampling area (such as dry or uncompacted leaves, branches, twigs, loose needles, etc.). Keep the litter layer, i.e. any layers which remain stuck to the ground. In habitats that have a lot of litter (such as tropical rainforests), collect the sample when there is 2 cm of litter or less above the soil layer. For peat habitats, start the sampling where the decomposed organic matter is smaller than 1 cm.

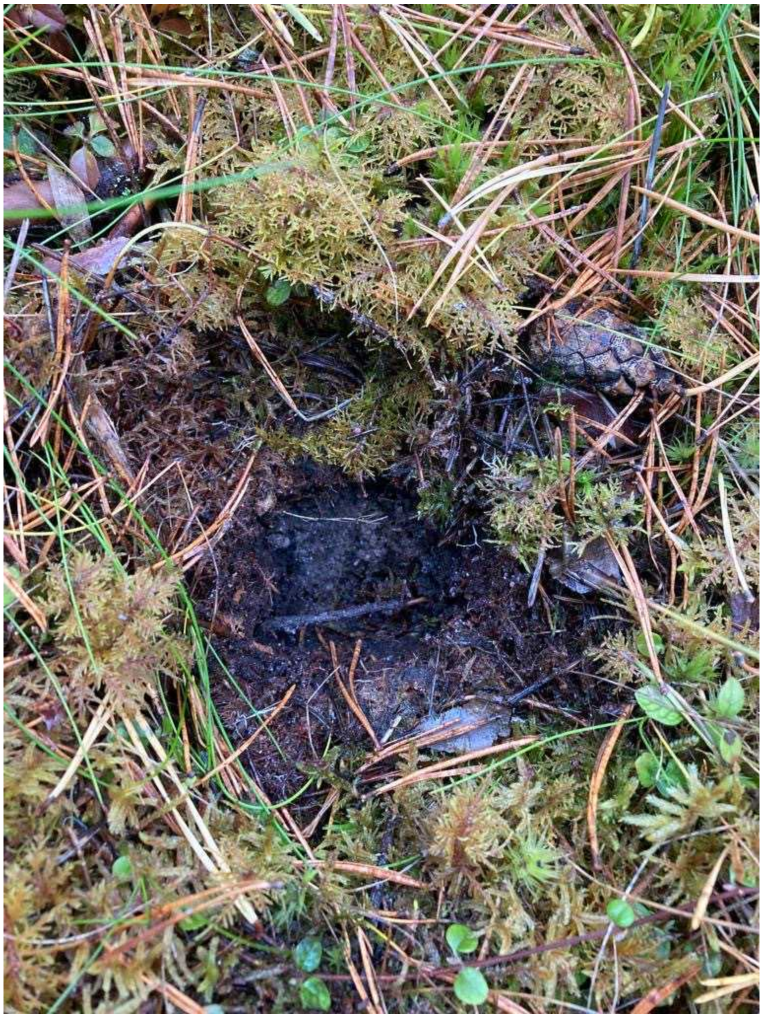

Remove the plant layer (and all green matter) from the area you are sampling.

6 Use a knife/spade to cut a 5 cm diameter hole. With a tablespoon or a spade, dig out the soil to a depth of 5 cm and place into a clear plastic bag. You can use a 5 cm corer if you have one, and take a 5 cm deep core.

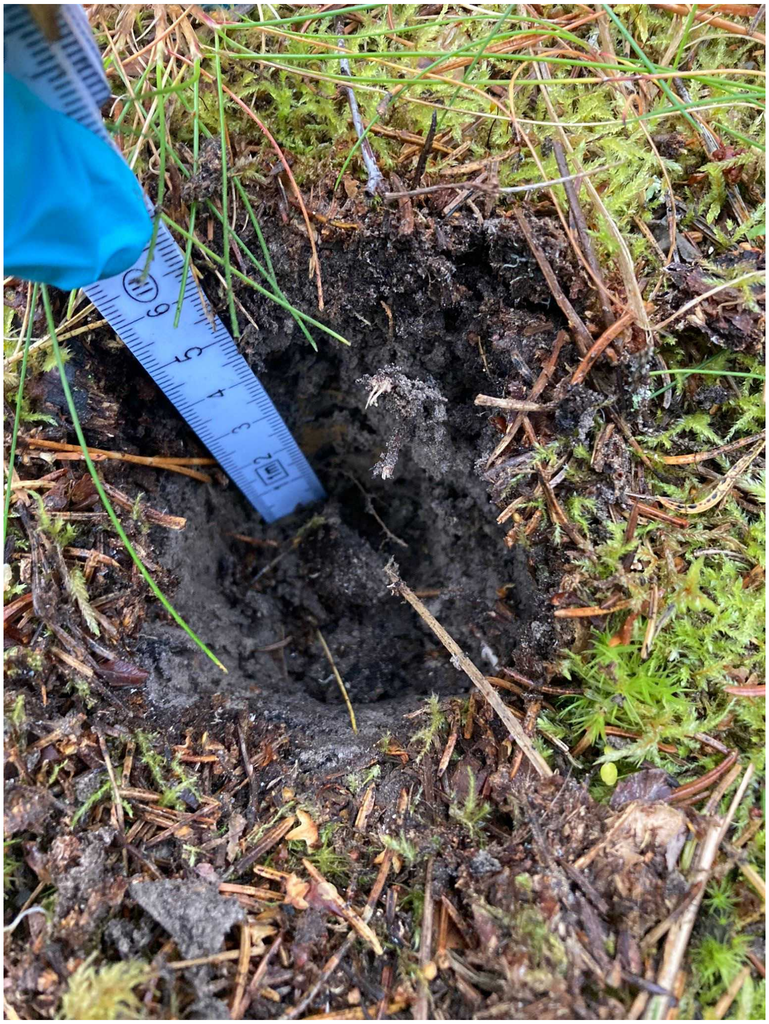

One replicate of soil is 5 cm in diameter and 5 cm in depth.

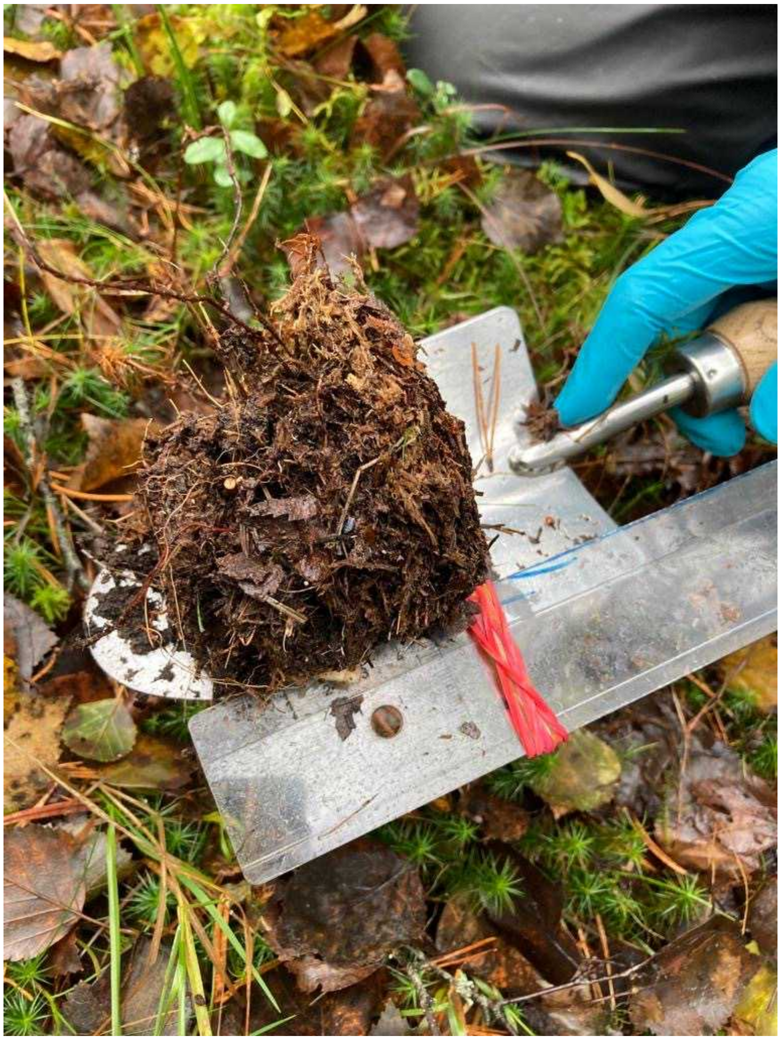

An example of one soil replicate (5 cm in diameter and 5 cm in depth).

7 Dig two more replicates between 1 and 2 metres from the first replicate, removing green plant parts as you go. Place all three replicates together in the same bag.

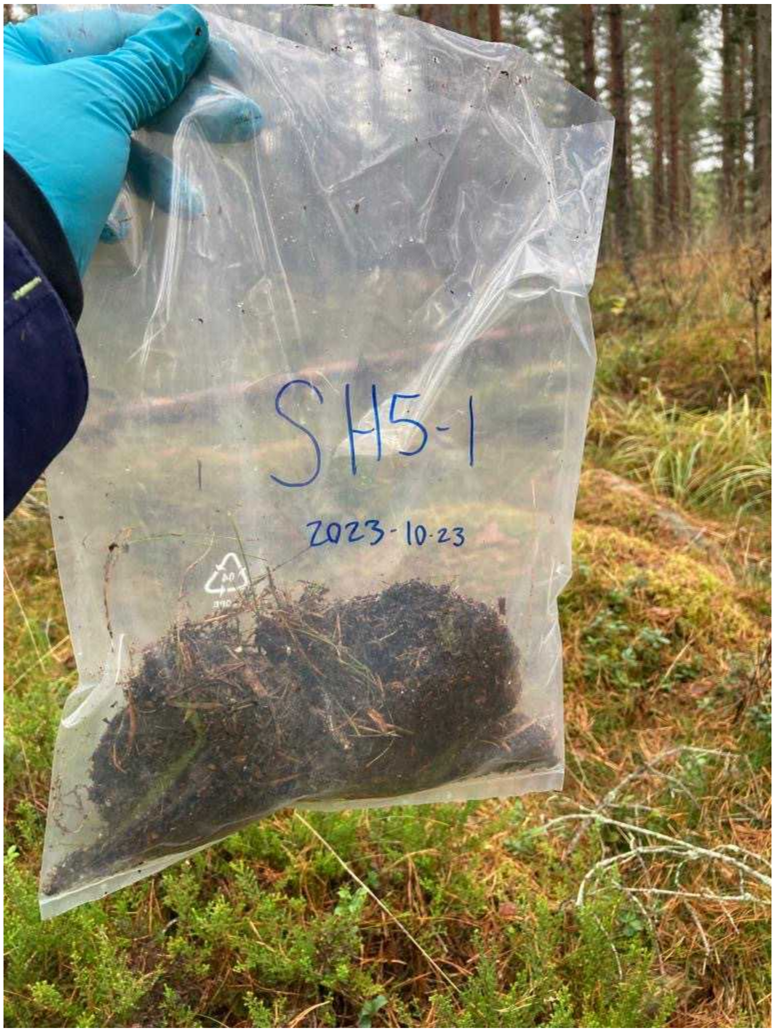

All three replicates comprise one soil sample, placed in a labelled bag ready to be mixed.

8 With gloved hands, remove the larger roots, leaves and branches that may have fallen into the bag. Do not tear a whole leaf into pieces, but do separate decomposed bits of litter and soil that may have aggregated together.

9 Mix the soil in the bag by holding it closed with one hand and scrunching the outside of the bag with the other hand to break down bigger soil or litter clumps.

10 Still with gloved hands, discard particles such as stones, litter, wood pieces and plant roots that are above 1 cm. 1m 30s
For soil samples containing a large amount of organic matter (such as peat), you may limit the removal process to 90 seconds.

The remaining soil can include organic matter with a particle size 1 cm or less.

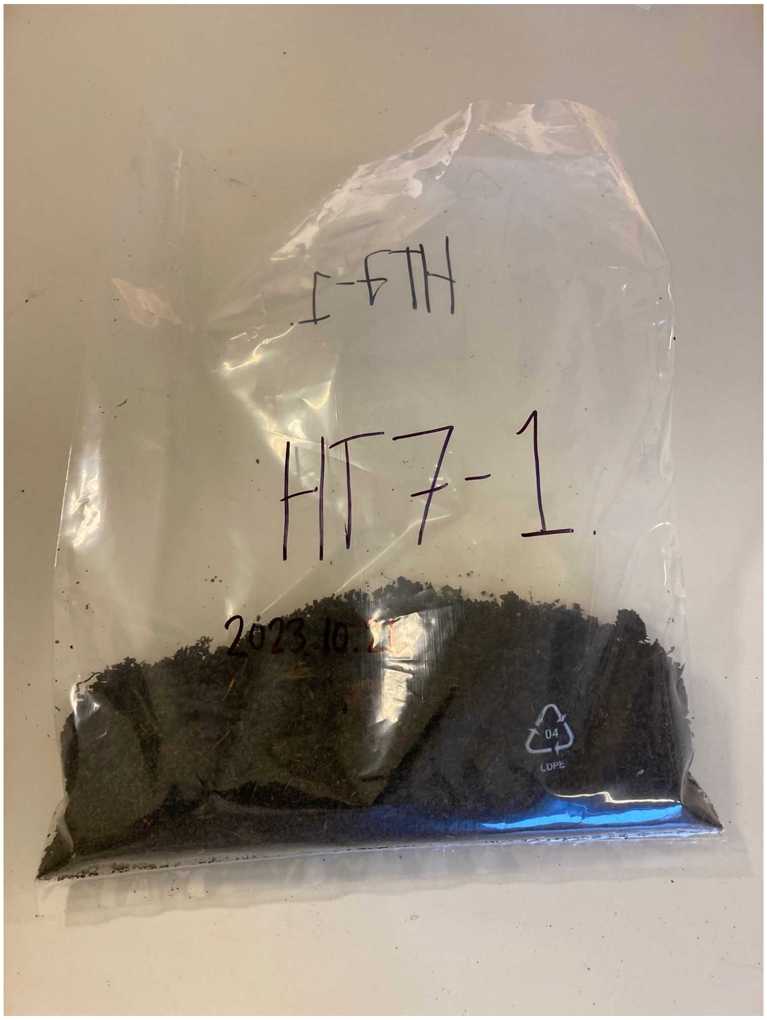

A soil sample after removal of larger particles.

11 Take three tablespoons of soil and place them into a paper bag. The soil should not contain any bigger (> 1 cm) particles, clumps or pieces.

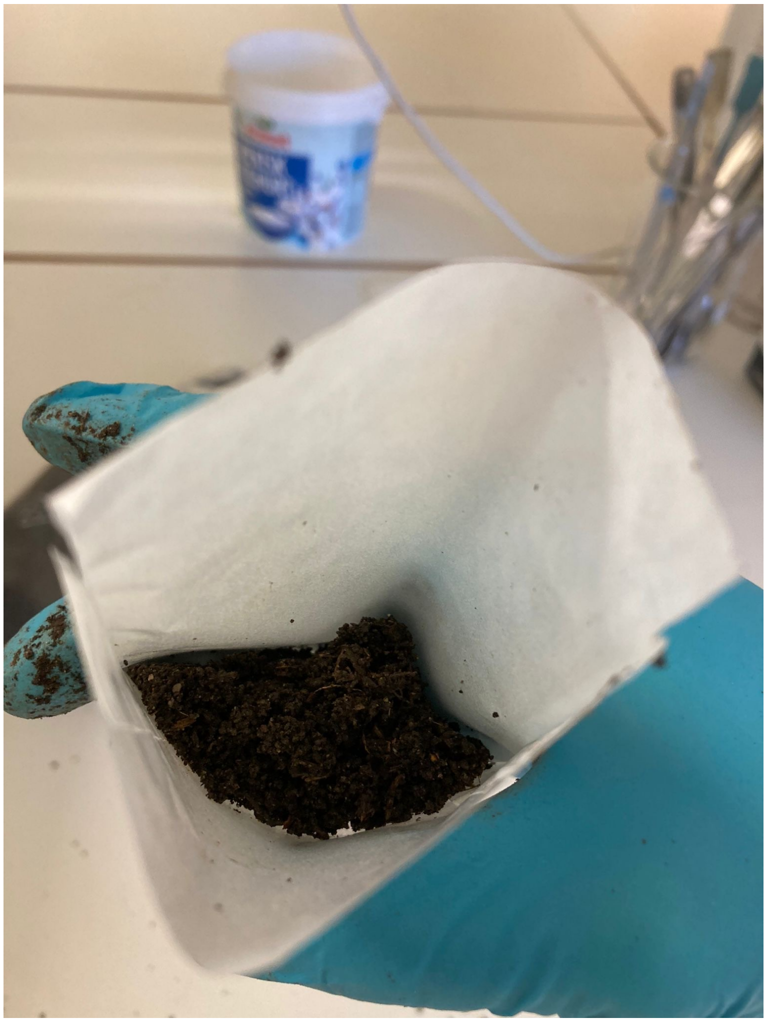

Three tablespoons of soil in a paper bag.

12 Clean the spoon between each sample with a disinfectant (flame, bleach, Virkon, etc.) and a sterile paper tissue (e.g. Kimtech).

13 Label the bag with a sample ID and other necessary information.

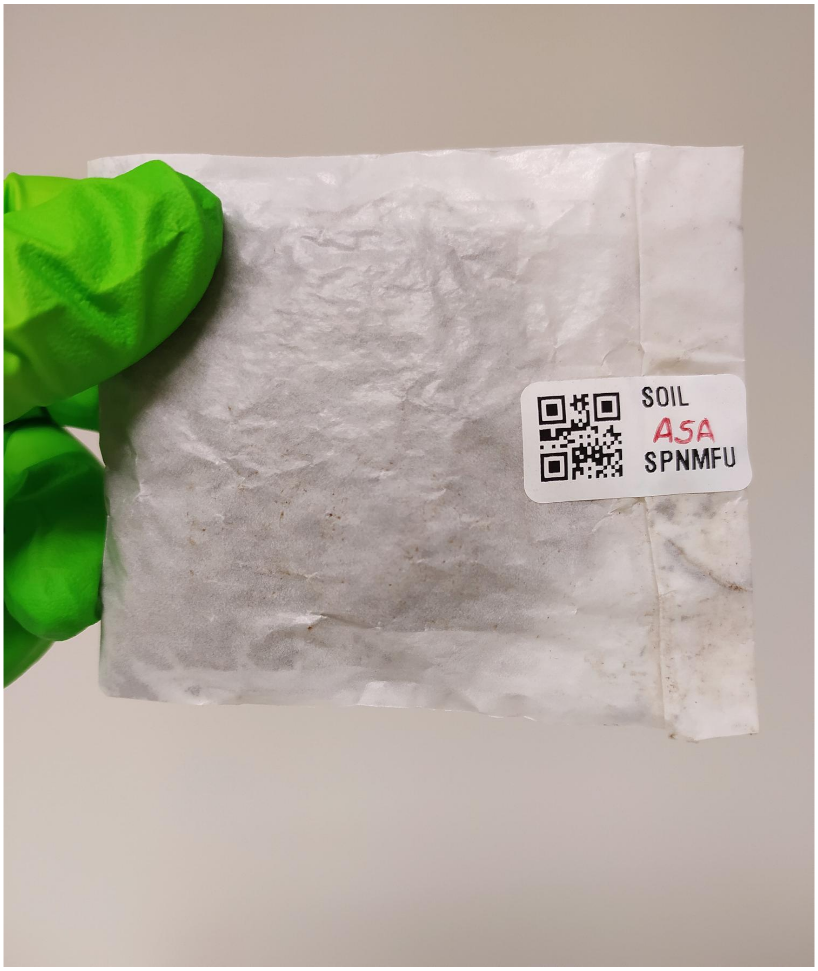

Labelled paper bag containing the final sample. The label associates the sample with the relevant metadata.

Note

Lifeplan teams use an app to scan the QR code and associate it to the matching corner number as defined in section 1.1. The sample ID is also associated with metadata including GPS location, date and time, name of collector and country of collection.

14 Repeat steps 3 to 13 for each camera/AudioMoth point in your site (i.e. three or five sets of samples). Make sure to put on new rubber gloves for each new sample (but not for each replicate).

15 Place each soil sample into its own resealable bag and close it tightly.

16 Place the samples in a cooler until you have returned to the laboratory

Soil Drying

17 Open each resealable sample bag and add some dry silica (approx. 50-100 grams). Flatten the bag to remove most of the air before sealing it. Leave to dry for approximately 24 hours.

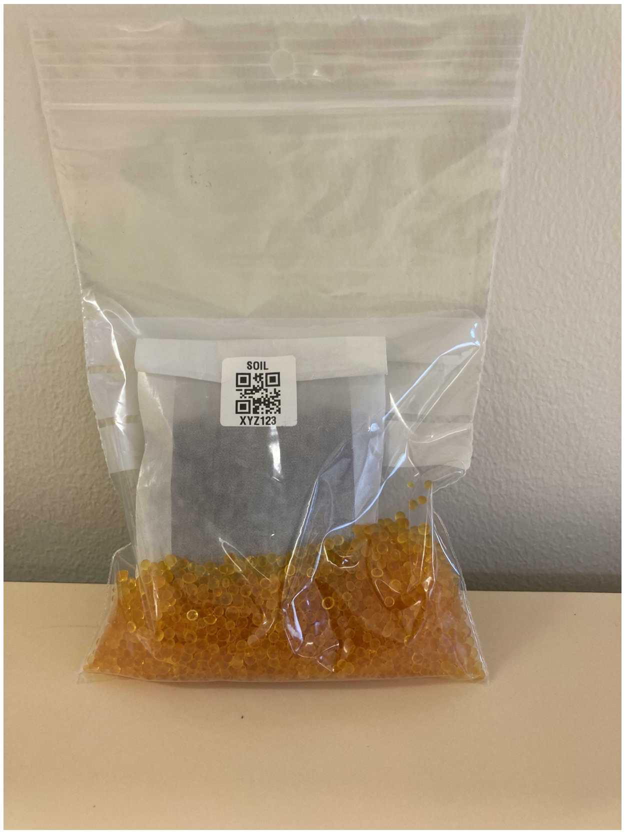

A soil sample with dry silica (orange). Note that the colours of silica may vary between brands.

18 When the silica changes colour, reopen the bag and swap out the wet silica for dry silica. Repeat
this every few days until the silica remains dry. How long this will take will very much depend on how humid your soil and environment is.

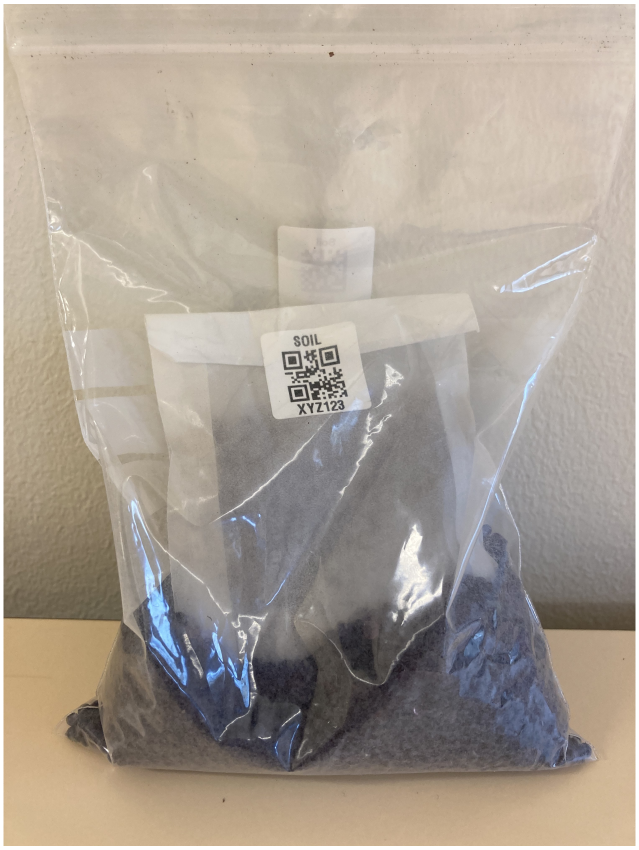

Soil sample with wet silica (blue).

19 When the silica remains dry, place the sample in a -20 °C freezer. Leave a few silica beads in the bag to ensure the environment remains dry until the samples can be freeze dried.

20 Freeze dry the soil samples.
In Lifeplan, the soil samples are freeze dried in a COOLSAFE 55-4 freeze dryer (Labogene) at -50 °C for at least 72 hours.

21 The used silica can be dried and re-used for other samples. To dry silica, place it in an oven or a drier at 100 °C until it changes to its dry colour, approximately 40 hours.
