# Supplementary material for: LIFEPLAN: A worldwide biodiversity sampling design
Source: PLoS One. 2024 Dec 31;19(12):e0313353. doi: 10.1371/journal.pone.0313353 (PMC11687782; doi:10.1371/journal.pone.0313353)
Supplement: Attachment — Submitted filename: Comments to authors.pdf [file pone.0313353.s006.pdf]

I have reviewed the manuscript titled “LIFEPLAN: A worldwide biodiversity sampling design” and I found it useful as well as interesting. As a biodiversity sampling design, I think this is timely and can be used broadly worldwide.

The only major comment I have is that the introduction doesn’t seem to address the main topic and the reader is not really “introduced” to the context. For example, the authors highlighted current issues with materials from long-standing process not being identified on time, and yet they are presenting another sampling design and not an identification method. By reading the current introduction, one could argue we don’t need additional sampling methods if we still have decades of samples to identify.

Obviously I agree this work is useful, and the authors should make it more clear in the introduction.

Another example, at lines **73-75,** this sentence may be a bit underappreciative of taxonomical efforts,

First of all, if we are talking about species identification only, this is an activity mostly performed by diagnosticians, who identify species (that have been described and discovered by others). The reason taxonomists are focused on smaller groups is due to the fact they also have to perform the species description, which is extremely time-consuming. This should be made abundantly clear in the introduction since your work focuses on collecting more specimens that will require taxonomists to study them if we want species to be identifiable.

I would suggest to replace the term “taxa” with “groups”, to make it clear you are not referring to species but larger taxonomical groups (e.g., families, superfamilies). Additionally, I would replace this sentence “and one human has a finite capacity for classification work” with something more on the line that we don’t have enough taxonomists available to cover all the taxonomical work required (this is a useful reference discussing this issue: https://doi.org/10.1093/zoolinnean/zlab072).

Overall, I would strongly suggest to redraft the introduction focusing on:

- Why do we need more **standardised** collection methods.
- What this method offers that other methods don’t.
- A bit more discussion on whether this new method could be use data from other methods. As it is more and more important to have long-standing studies to record biodiversity, one could argue that yet a another method (also quite expensive) may not be the best choice if instead we could adapt other methods/protocols that have already been implemented in the past few deacades.

Minor suggestions/corrections:

Line 50: You should be more consistent throughout the MS and decide if you want to use the capital letter (line 178) or not (line 50) for “national” and “nested”. Since you are using these terms as “objects” of your analysis, I would suggest to use a capital letter but to define the concept here.

Lines 75-78: Following to my comment above, please, ensure the references mentioned here [7-8] did not also described new species, as there is a clear difference between species identification and species delimitation.

Line 123: Perhaps replace “foci” with “targets”, as the former is not really commonly used.

Line 154: Could you please list the Nordic countries? Even in brackets after the first mention.

Line 158: I think this is the best place to explain a bit better what you mean with “national”, “nested”.

Line 171: either remove capital letter from “Natural” or make “Urban” the same.

Line 192: not exactly sure what you mean with “realised” here. Could you please clarify or use a synonym?

Line 223: Replace “have” with “had” for consistency.

Line 226: Apologies, but I still don’t understand the meaning of “realised” in this context.. See comment above.

Line 266: “growing season” of what?

Line 305: Please, italicise all genes’ names.

Line 309: Add degree symbol.
